# Cinnamyl-Modified Polyglycidol/Poly(ε-Caprolactone) Block Copolymer Nanocarriers for Enhanced Encapsulation and Prolonged Release of Cannabidiol

**DOI:** 10.3390/pharmaceutics15082128

**Published:** 2023-08-13

**Authors:** Natalia Toncheva-Moncheva, Erik Dimitrov, Georgi Grancharov, Denitsa Momekova, Petar Petrov, Stanislav Rangelov

**Affiliations:** 1 Institute of Polymers, Bulgarian Academy of Sciences, “Akad. G. Bonchev” Street., bl. 103A, 1113 Sofia, Bulgaria; e_dimitrov@polymer.bas.bg (E.D.); granchar@polymer.bas.bg (G.G.); ppetrov@polymer.bas.bg (P.P.); 2 Department of Pharmaceutical Technology and Biopharmaceutics, Faculty of Pharmacy, Medical University-Sofia, 2 Dunav Street, 1000 Sofia, Bulgaria; dmomekova@pharmfac.mu-sofia.bg

**Keywords:** click reactions, polyglycidol, poly(ε-caprolactone), self-assembly, cannabidiol, functional nanomaterials

## Abstract

The present study describes the development of novel block copolymer nanocarriers of the phytocannabinoid cannabidiol (CBD), designed to enhance the solubility of the drug in water while achieving high encapsulation efficiency and prolonged drug release. Firstly, a well-defined amphiphilic block copolymer consisting of two outer hydrophilic polyglycidol (PG) blocks and a middle hydrophobic block of poly(ε-caprolactone) bearing pendant cinnamyl moieties (P(CyCL-*co*-CL)) were synthesized by the click coupling reaction of PG-monoalkyne and P(CyCL-*co*-CL)-diazide functional macroreagents. A non-modified polyglycidol/poly(ε-caprolactone) amphiphilic block copolymer was obtained as a referent system. Micellar carriers based on the two block copolymers were formed via the solvent evaporation method and loaded with CBD following two different protocols—loading during micelle formation and loading into preformed micelles. The key parameters/characteristics of blank and CBD-loaded micelles such as size, size distribution, zeta potential, molar mass, critical micelle concentration, morphology, and encapsulation efficiency were determined by using dynamic and static multiangle and electrophoretic light scattering, transmission electron microscopy, and atomic force microscopy. Embedding CBD into the micellar carriers affected their hydrodynamic radii to some extent, while the spherical morphology of particles was not changed. The nanoformulation based on the copolymer bearing cinnamyl moieties possessed significantly higher encapsulation efficiency and a slower rate of drug release than the non-modified copolymer. The comparative assessment of the antiproliferative effect of micellar CBD vs. the free drug against the acute myeloid leukemia-derived HL-60 cell line and Sezary Syndrome HUT-78 demonstrated that the newly developed systems have pronounced antitumor activity.

## 1. Introduction

Nanoscopic polymer-based drug-delivery systems are rapidly entering the pharmaceutical field since they are beneficial for treating severe human diseases. Such systems enable the introduction of a variety of therapeutic substances into the body and improve their efficacy and safety profiles [1,2]. Despite their vast potential, the key characteristics of the polymer-based nanoformulations still need improvement to achieve superior colloidal stability, protection from enzyme actions, and diminished particle interactions with plasma proteins, which could guarantee a prolonged circulation in the bloodstream, favorable pharmacokinetic profile, and enhanced bioavailability [3,4].

Polymer micelles are core-corona aggregates mainly formed in an aqueous environment by the self-assembly of amphiphilic block copolymers. Currently, they are among the most-important nanosized carriers used for the solubilization and delivery of poorly water-soluble drugs [5,6]. The hydrophobic moieties of the copolymer build the micellar core, where drug molecules are embedded, while the corona-forming hydrophilic moieties act as a stabilizer against particle agglomeration and protect the payload from enzymatic attack [7,8].

Poly(ethylene oxide) (PEO), poly(vinyl alcohol) (PVA), and polyvinylpyrrolidone (PVP) are commonly used as corona-forming hydrophilic components of the copolymer micelles [9,10,11]. The polymers often used as core-forming hydrophobic blocks include poly(propylene oxide) (PPO), poly(ε-caprolactone) (PCL), poly(lactic acid) (PLA), polyglycolide (PGA), poly(D,L-lactide-*co*-glycolide) (PLGA), and poly(aspartic acid) (PASA) [12,13,14,15,16,17]. The aliphatic polyesters PCL, PLA, and PGA, and their copolymers are biocompatible and biodegradable and therefore are preferred in the preparation of nanosized drug carriers. In particular, PCL is characterized by high permeability to small drug molecules and a negligible tendency to generate an acidic environment during degradation as compared to PLA and PGA [18]. In addition, due to the semicrystalline nature of PCL, the micelles possess superior structural stability (frozen micelles) and retain their integrity upon injection into the bloodstream, regardless of severe dilution. Thus, the probability of delivering the drug to the target site is improved.

The significant progress in polymer synthesis in the last few decades has enabled the design of novel (co)polymers with controlled composition, molar mass, topology, and well-defined positions of the functional groups along the chain [19,20]. For example, the covalent attachment of different ligands to the hydrophilic corona-forming segments allows for the better internalization of the nanocarrier by the tumor cells, thus eliminating the systemic side effects of antineoplastic drugs and hypersensitization toward multidrug resistance. On the other hand, introducing specific pendant groups or segments into the hydrophobic blocks increases the compatibility between the micellar core and the hydrophobic drugs, resulting in enhanced drug-loading efficiency [21,22,23].

Different studies on the effect of modifying amphiphilic copolymers comprising PEO, methoxypolyoxyethylene (MPEO), PLA, PLGA, PCL, or poly(amino acid) (PAA) with “compatibilizing” pendant groups demonstrate that the properties of the nanocarriers can be properly modulated to achieve a more-efficient loading of drugs compared to the nonmodified systems, and also to avoid unwanted burst release [23,24,25,26,27,28,29,30,31,32].

PEO is extensively used in many conventional medical and cosmetic formulations, cancer treatment products, and polymer-mediated gene delivery systems [33,34], due to its relative inertness and stealth properties. Such a ubiquity of PEO, however, could be problematic and cause potential safety issues such as antibody formation, hypersensitivity, and vacuolation, which might limit its application in products intended for the treatment of patients [35,36,37].

One promising candidate for the replacement of PEO is polyglycidol (PG). Polyglycidol (also known as polyglycerol) is a hydrophilic biocompatible polyether with a main chain structure similar to that of PEO [38]. They differ in that PG bears a hydroxymethylene group in each repeating monomer unit that not only modulates the solubility properties of this polymer but also allows for the further chemical modification of PG-based macrochains and nanocarriers. Recent studies have disclosed the positive effect of the hydrophilic PG-based corona on micelle stability and therapeutic efficacy, thus demonstrating the outstanding potential of PG as a PEO alternative for the half-life extension of therapeutics [39,40,41,42,43]. 

Linear PG can be obtained via a two-step procedure involving the ring-opening anionic polymerization of protected glycidols (glycidyl ethers, the hydroxyl groups of which are protected by a suitable group) followed by a deprotection reaction [44,45,46,47]. Moreover, various PG-based linear amphiphilic AB, ABA, and ABC block co- and terpolymers as well as other non-linear architectures can be synthesized by the covalent linking of protected PG to other preformed polymers by, for instance, employing the click chemistry technique, and the subsequent cleavage of the protective groups [48,49].

Cannabidiol (CBD) is a phytocannabinoid with well-known beneficial pharmacological effects, as well as analgesic and anti-inflammatory activities mediated by the inhibition of cyclooxygenase and lipoxygenase (ESI Scheme S1) [50,51,52]. However, the poor solubility of CBD in aqueous solutions significantly decreases its bioavailability and favorable biodistribution. To address this issue, we designed novel amphiphilic block copolymer nanocarriers for the controlled delivery of CBD, which can solubilize the drug in physiological media and ensure a sustained release profile. In this study, a polyglycidol-*block*-poly(propylene oxide)-block-poly(α-cinnamyl-*ε*-caprolactone-*co*-ε-caprolactone)-*block*-poly(propylene oxide)-block-polyglycidol [PG_50_-b-PPO_4_-b-[P(CyCL)_4_-co-(CL)_40_]-b-PPO_4_-b-PG_50_] copolymer, comprising a hydrophobic middle block bearing pendant cinnamyl moieties and a non-modified PG_45_-*b*-PCL_35_-*b*-PG_45_ copolymer were synthesized and used for preparing micellar nanocarriers. The blank and CBD-loaded micelles were characterized, and their key parameters/characteristics such as size, size distribution, zeta potential, molar mass, morphology, and encapsulation efficiency were determined. The cytotoxicity, in vitro release profile, and antitumor activity of the systems based on modified and non-modified copolymers were assessed as well.

## 2. Materials and Methods

### 2.1. Materials

The two polymer precursors (N_3_-[(PCyCL)_4_-*co*-(PCL)_40_]-N_3_ and PEEGE_50_-*b*-PPO_4_-C≡CH) as well as the referent copolymer (PG_45_-*b*-PCL_35_-*b*-PG_45_) were prepared as described elsewhere [48,53]. Synthetic details and characterization data are presented in the ESI (Schemes S2 and S3, Figures S1 and S2). Ethoxyethyl glycidyl ether (EEGE) was synthesized as described elsewhere [44,48]. Methanol (ACS reagent, ≥99.8%, Sigma-Aldrich, Burlington, MA, USA) was used as received. Tetrahydrofuran (THF, >99.5% Fisher Scientific, Waltham, MA, USA) was dried by refluxing over a sodium-benzophenone mixture and subsequently distilled. Copper (I) bromide (99.999% trace metals basis, Sigma-Aldrich, Burlington, MA, USA), N,N,N′,N″,N″-pentamethyldiethylenetriamine (PMDETA, 99%, Sigma-Aldrich, Burlington, MA, USA), AlCl_3_·6H_2_O (99%, Sigma-Aldrich, St. Louis, MO, USA), 1,6-diphenyl-1,3,5-hexatriene (DPH, Sigma-Aldrich, St. Louis, MO, USA), and phosphate-buffered saline (PBS) were used as received. Cannabidiol was kindly donated by PBG GLOBAL LTD., Sofia, Bulgaria, and used without further purification. Deionized water was obtained using a Millipore MilliQ system (Merck, Darmstadt, Germany) and additionally filtered through a 220 nm PTFE filter and a 20 nm cellulose filter. The antiproliferative activity of free and micellar CBD was evaluated against the two human tumor cell lines, namely: acute myelocyte leukemia-derived cells (HL-60) and cutaneous T lymphocytes (HUT-78—Sezary Syndrome). The cell lines were purchased from the German Collection of Microorganisms and Cell Cultures (DSMZ GmbH, Braunschweig, Germany) and were cultivated in a growth medium based on 90% RPMI-1640 and 10% fetal bovine serum (FBS) under standard conditions of 37 °C and a 5% humidified CO_2_ atmosphere. Only cells growing in the exponential phase were used.

### 2.2. Synthesis of Polyglycidol-Block-Poly(Propylene Oxide)-Block-Poly(α-Cinnamyl-ɛ-Caprolactone-co-ɛ-Caprolactone)-Block-Poly(Propylene Oxide)-Block-Polyglycidol Block Copolymer

The polymer precursor N_3_-[(PCyCL)_4_-*co*-(PCL)_40_]-N_3_ (0.076 g, 0.0154 mmol, 1 eq) and CuBr (0.0220 g, 0.154 mmol, 10 eq) were added to a 50 mL round-bottom flask under an argon atmosphere. Dry THF (3 mL) was added via a syringe, and the solution was purged with argon and stirred vigorously for 20 min. Monoalkyne-terminated PEEGE_50_-*b*-PPO_4_-C≡CH (0.247 g, 0.03087 mmol, 2 eq) was dissolved in dry THF (2 mL) and added to the solution along with PMDETA (0.0396 g, 0.228 mmol, 20 eq). The click coupling reaction was carried out at 30 °C for 24 h. The reaction mixture was cooled to RT, diluted with THF (30 mL), and filtered through a column filled with neutral alumina to remove copper complexes. The excess THF was evaporated; the crude product was dissolved in methanol (10 mL) and dialyzed against a methanol/water mixture (10:1 v/v, membrane, MWCO 8 kDa) for 72 h. The methanol was removed using a rotary vacuum evaporator, and the copolymer was recovered by freeze-drying. Yield: 0.258 g (80%); M_n_ = 15,000 g·mol^−1^, M_w_/M_n_ = 1.3.

PEEGE blocks were derivatized into PG ones by treatment with AlCl_3_·6H_2_O, as described elsewhere [54,55]. PEEGE_50_-*b*-(PO_4_)-*b*-[(PCyCL)_4_-*co*-(PCL)_40_]-*b*-(PO_4_)-*b*-PEEGE_50_] (0.100 g, 1 eq) was dissolved in methanol (8.9 mL, 3200 eq) at 40 °C, and then AlCl_3_·6H_2_O (0.020 g, 10 eq) was added under stirring. The hydrolysis was conducted at the same temperature for 48 h until the complete disappearance of the methine proton signal at 4.75 ppm in the ^1^H-NMR spectrum. The reaction mixture was filtered through Hyflo Super Cel diatomaceous earth, and then methanol was evaporated under reduced pressure.

### 2.3. Preparation of Polymeric Micelles

A copolymer solution (5 mg of copolymer in 5 mL of methanol) was added dropwise to purified water (5 mL) at room temperature under vigorous stirring (1000 rpm). After 30 min, the organic solvent was evaporated under reduced pressure at 37 °C to obtain a slightly opalescent, colorless micellar dispersion with a concentration of 1 mg·mL^−1^.

### 2.4. Loading of CBD

**Scheme 1:**
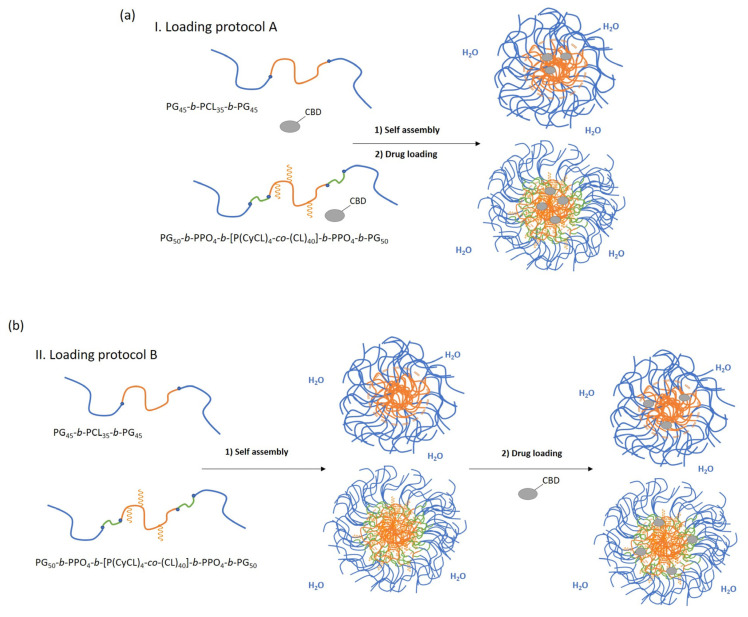
Preparation of CBD-loaded polymeric micelles according to protocol A (**a**) and protocol B (**b**).

The micelles of the novel (PG_50_-b-PPO_4_-b-[P(CyCL)_4_-co-(CL)_40_]-b-PPO_4_-b-PG_50_) and referent (PG_45_-b-PCL_35_-b-PG_45_) copolymers were loaded with CBD according to two protocols, schematically depicted in Scheme 1. The encapsulation efficiency (EE) was calculated from the following equation:

(1) $$EE\left(\%\right)=\frac{Total\;mass\;of\;CBD-Mass\;of\;free\;CBD}{Total\;mass\;of\;CBD}\times 100$$

Protocol A: The selected copolymer (5 mg) and CBD (0.5 mg) were dissolved in methanol (5 mL) at a copolymer/CBD-mass ratio of 10:1. After that, the organic solution was added dropwise to purified water (5 mL) at room temperature under vigorous stirring (1000 rpm). The resulting mixture was stirred additionally for 30 min at the same temperature. Finally, the organic solvent was evaporated by a rotary vacuum evaporator at 37 °C, yielding a slightly opalescent, colorless aqueous micellar dispersion. The dispersion was filtered (Nylon, 0.22 μm) and the filter was rinsed with ethanol. The collected filter fraction was quantified by spectrophotometric measurements (λ = 274 nm) to determine the mass of free, unentrapped CBD.

Protocol B: The selected copolymer (5 mg) was dissolved in methanol (5 mL). After that, the organic solution was added dropwise to purified water (5 mL) at room temperature under vigorous stirring (1000 rpm). The resulting mixture was stirred additionally for 30 min at the same temperature. Next, the organic solvent was evaporated by a rotary vacuum evaporator at 37 °C. The CBD (0.5 mg) (copolymer/CBD-mass ratio 10:1) dissolved in 40 µL methanol was added to the as-prepared micellar dispersion. The resulting mixture was stirred additionally for 30 min at the same temperature. Finally, the traces of organic solvent were evaporated under argon, yielding a slightly opalescent, colorless aqueous micellar dispersion. The dispersion was filtered (Nylon, 0.22 μm) and the filter was rinsed with ethanol. The collected filter fraction was quantified by spectrophotometric measurements (λ = 274 nm) to determine the mass of free, unentrapped CBD.

### 2.5. Proton Nuclear Magnetic Resonance (^1^H-NMR)

^1^H-NMR measurements were conducted on a Bruker Avance II spectrometer operating at 600 MHz using CDCl_3_, DMSO-d_6,_ or CD_3_OD at 25 °C. 

### 2.6. Size Exclusion Chromatography (SEC)

Analyses were performed on Shimadzu Nexera HPLC chromatograph, equipped with a degasser, a pump, an auto-sampler, a RI detector, and three columns: 10 µm PL gel mixed-B, 5 µm PL gel 500 Å, and 50 Å. THF was used as the eluent at a flow rate of 1.0 mL·min^−1^ and a temperature of 40 °C. The sample concentration was 1 mg·mL^−1^ and GPC was calibrated with polystyrene standards. 

### 2.7. Transmission Electron Microscopy (TEM)

Micrographs were obtained using an HRTEM JEOL JEM-2100 transmission electron microscope operating at 200 kV. The samples were prepared by depositing a drop of the dispersions onto a carbon grid and the subsequent evaporation of the solvent under a vacuum. To visualize the hydrophilic corona with high contrast, a uranyl acetate staining protocol prior to sample preparation was utilized [56].

### 2.8. Atomic Force Microscopy (AFM)

The images were obtained using a Bruker Dimension Icon Instrument operating at a 1.00 Hz scan rate under ambient conditions. Moreover, 2 μL of the copolymer dispersions was placed onto a freshly cleaned glass substrate (1 cm^2^) and spin-casted at 2000 rpm for a minute. AFM measurements were performed in ScanAsyst (Peak Force Tapping) mode.

### 2.9. Dynamic and Electrophoretic Light Scattering

The preliminary assessment of the particle size and size distribution was carried out by dynamic light scattering (DLS) measurements on a NanoBrook 90 Plus PALS instrument (Brookhaven Instruments Corporation), equipped with a 35 mW red diode laser (λ = 660 nm) at a scattering angle of 90 °. The measurements were taken at 25 °C and 37 °C applying a dust cut-off of 30 micellar dispersions at concentrations of 1 mg·mL^−1^ and fixed volumes of 1.7 mL.

The apparent hydrodynamic radii (R_h_^90^) were determined according to the Stokes–Einstein equation:

R_h_^90^ = kT/(6πηD_90_) (2)

where k is the Boltzmann constant, η is the solvent viscosity at temperature T in Kelvin and D_90_ is the diffusion coefficient measured at an angle of 90°. Each measurement was performed in triplicate. 

The electrophoretic light scattering measurements were carried out on the same instrument at a scattering angle of 15° and 25 °C and 37 °C. The principle of phase analysis light scattering (PALS) was applied for the measurements of electrophoretic mobility. The ζ potentials were calculated using the Smoluchowski equation:

ζ = 4πηυ/ε(3)

where η is the solvent viscosity, υ is the electrophoretic mobility, and ε is the dielectric constant of the solvent. 

### 2.10. Multiangle Dynamic and Static Light Scattering

Dynamic light scattering measurements were performed on a Brookhaven BI-200 goniometer with vertically polarized incident light at a wavelength λ = 633 nm supplied by a He–Ne laser operating at 35 mW and equipped with a Brookhaven BI-9000 AT digital autocorrelator. The scattered light was measured for dilute aqueous dispersions of the empty and CBD-loaded micellar dispersions in the concentration range 0.417–1.0 mg·mL^−1^ at 25 °C. Measurements were made at angles θ in the range of 50–130°. The autocorrelation functions were analyzed using the constrained regularized algorithm CONTIN [57] to obtain the distributions of the relaxation rates (Γ). The latter provided distributions of the apparent diffusion coefficient (D = Γ/q^2^), where q is the magnitude of the scattering vector given by q = (4πn/λ)sin(θ/2), n is the refractive index of the medium. The mean hydrodynamic radius was obtained by the Stokes–Einstein equation:

R_h_ = kT/(6πηD_0_) (4)

where k is the Boltzmann constant, η is the solvent viscosity at temperature T in Kelvin, and D_0_ is the diffusion coefficient at infinite dilution.

Static light scattering (SLS) measurements were carried out in the interval of angles from 40 to 140° at 25 °C using the same light scattering set. The SLS data were analyzed using the Zimm plot software provided by Brookhaven Instruments. Information on the weight-average molar mass, M_w_, the radius of gyration, R_g_, and the second virial coefficient, A_2_, was obtained from the dependence of the quantity (Kc/R_θ_) on the concentration (c) and scattering angle (θ). Here, K is the optical constant given by K = 4π^2^n_0_^2^(dn/dc)^2^/N_A_λ^4^, where n_0_ is the refractive index of the solvent, N_A_ is Avogadro’s constant, λ is the laser wavelength, and R_θ_ is the Rayleigh ratio at angle θ. dn/dc is the refractive index increment measured in water in separate experiments on an Orange GPC19 DNDC refractometer. The dn/dc values of the investigated systems were in the 0.131–0.133 g·mL^−1^ range. The DLS and SLS measurements were performed at 25 and 37 °C.

### 2.11. Spectrophotometric Determination of the Critical Micelle Concentration (CMC)

Furthermore, 20 μL of a 0.4 mM solution of 1,6-diphenyl-1,3,5-hexatriene (DPH) in methanol was added to 2 mL micellar dispersions with increasing concentrations in the range 9.765 × 10^−4^–1.0 mg·mL^−1^. The samples were incubated in the dark for 24 h at room temperature. UV-vis absorption spectra of DPH in the wavelength interval λ = 300–500 nm at room temperature were recorded on a Beckman Coulter DU 800 UV-vis spectrometer. The intensities of the absorption peak at 356 nm were plotted against the polymer concentration. The CMC value was determined as the break in the absorbance intensity versus the concentration curve.

### 2.12. Drug-Release Study

The release of CBD was evaluated as a function of time using the dialysis method [58]. In short, 2 mL of tested formulations was inserted into a dialysis bag (MWCO 12,000–14,000, Sigma-Aldrich, Steinheim, Germany) and placed in a 50 mL dissolution medium of phosphate-buffered saline pH 7.4 and 10% ethanol to retain the solubility of the released CBD. The acceptor medium was in constant motion (200 rpm) and the circulating water jacket (Huber, Germany) maintained the temperature at 37 ± 0.5 °C during the study. At predetermined time points, 1 mL samples from the released medium were withdrawn and analyzed by UV–vis spectroscopy at 274 nm using a calibration curve with linearity in the concentration range of 0.0025 to 10 µg·mL^−1^ (correlation coefficient R^2^ = 0.995). The aliquots were replaced with equal volumes of fresh medium. The drug-release studies were carried out threefold.

### 2.13. Cytotoxicity Assessment

The cytotoxicity of the copolymers and the micelles prepared thereof was evaluated by standard MTT-dye reduction assay. The assay is based on the aptitude of the mitochondrial succinate dehydrogenase of viable cells to metabolize the yellow tetrazolium MTT dye to a violet formazan. The experiment was performed as described by Mosman [59] with small modifications [60]. Cells in the exponential phase were seeded in 96-well microplates (100 μL/well) at a density of 1 × 10^5^ cells·mL^−1^ and incubated at 37 °C for 24 h. Afterward, they were exposed to various concentrations of empty micelles or free or micellar CBD for a period of 72 h. For each test group, a set of at least eight wells were used. After the exposure time, to each well aliquots of 10 μL of MTT solution (10 mg·mL^−1^ in PBS) was added. Next, the microplates were incubated for 4 h at 37 °C and the MTT-formazan crystals formed were dissolved by the addition of 100 μL/well of 5% formic acid solution in 2-propanol. The MTT-formazan absorption was recorded using a Beckman–Coulter DTX800 multimode microplate reader at 580 nm. Thereafter the cell survival fractions were calculated as a percentage of the untreated control. In addition, IC_50_ values were derived from the concentration-response curves.

### 2.14. Statistical Analysis

The data are presented as the mean standard deviation (SD) of three independent experiments. The correlation coefficients for the linear sections of the curves were in the 0.992–0.999 range.

## 3. Results and Discussion

### 3.1. Copolymer Synthesis and Characterization

**Scheme 2:**
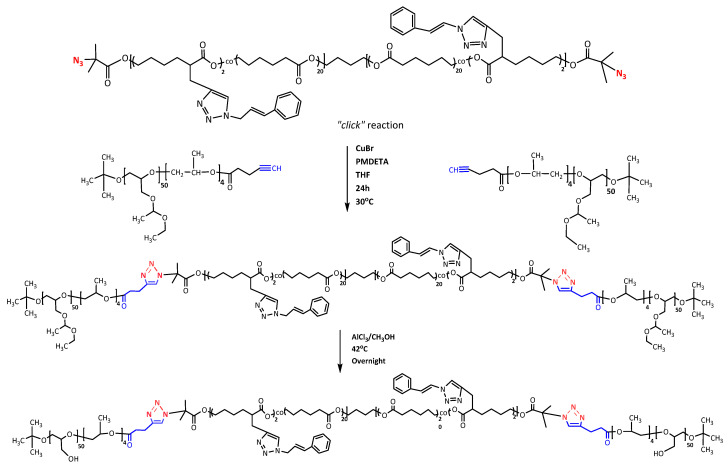
Schematic representation of the synthesis of PEEGE_50_-*b*-PPO_4_-*b*-[P(CyCL)_4_-*co*-(CL)_40_]-*b*-PPO_4_-*b*-PEEGE_50_ copolymer by copper-catalyzed “click” coupling reaction and subsequent deprotection leading to the amphiphilic PG_50_*-b*-PPO_4_-*b*-[P(CyCL)_4_-*co*-(CL)_40_]-*b*-PPO_4_-*b*-PG_50_ block copolymer.

The synthetic approach to preparing the novel copolymer is based on a click coupling reaction of appropriately functionalized polymer intermediates. The reaction steps are presented in Scheme 2. Firstly, a PCL-based polymer bearing pendant cinnamyl groups was prepared by a procedure described elsewhere [53] and functionalized with terminal azide groups. The reaction scheme, molar mass characteristics, and composition of this polymer precursor are presented in the ESI. Separately, a monoalkyne functionalized PEEGE was obtained by the ring-opening polymerization of EEGE. Upon completion of the polymerization, a short spacer of four oxypropylene units was introduced to facilitate the subsequent modification of the as-prepared monohydroxy prepolymer with a clickable alkyne end group via esterification with 4-pentynoic acid [48]. A detailed reaction scheme as well as SEC curves and characterization data (^1^H NMR spectra) are presented in the ESI.

**Figure 1:**
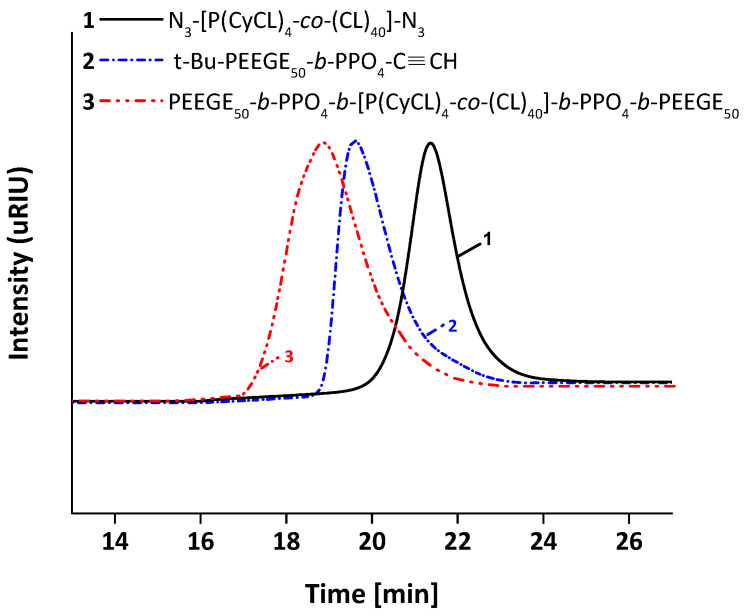
SEC traces of the polymer precursors tBu-PEEGE_50_-*b*-PPO_4_-C≡CH and N_3_-[P(CyCL)_4_-*co*-(CL)_40_]-N_3_), and the resulting copolymer PEEGE_50_-*b*-PPO_4_-*b*-[P(CyCL)_4_-*co*-(CL)_40_]-*b*-PPO_4_-*b*-PEEGE_50_. THF was used as the eluent at a flow rate of 1.0 mL·min^−1^, at a temperature of 40 °C.

The azide-alkyne click reaction was carried out in THF at 30 °C for 24 h using a CuBr/PMDETA catalytic complex (Scheme 2). SEC and ^1^H-NMR analyses were used to characterize the product (Figure 1 and Figure S3). SEC showed a clear shift to lower retention times (corresponding to higher molar mass) of the product in comparison to the PEEGE- and PCL-based prepolymers, while maintaining a monomodal and narrow (M_w_/M_n_ = 1.3) molar mass distribution (Figure 1). Furthermore, in the ^1^H-NMR spectrum (Figure S3a), all proton signals characteristic for the two precursors were evident, whereas the signals assigned to the alkyne protons at 2.01 ppm disappeared and a new signal for the methyne protons of the triazole ring at 8.1 ppm appeared. In the final step, the copolymer was treated with AlCl_3_.6H_2_O to remove the protective EEGE groups and to convert the flanking PEEGE blocks into blocks of linear polyglycidol (Scheme 2). The ^1^H-NMR spectrum of the final product is presented in Figure S3b. The disappearance of the signal for the methyl protons of the protective EEGE groups and the appearance of the new signal assigned to OH groups of PG at 4.46 ppm proved the effective release. The results suggested the high efficiency of both the click reaction for coupling of the prepolymers and the modification reaction to release the protective groups. The composition and molar mass characteristics of the novel and referent copolymers are presented in Table 1. 

### 3.2. Preparation of Blank and CBD-Loaded Polymeric Micelles

**Figure 2:**
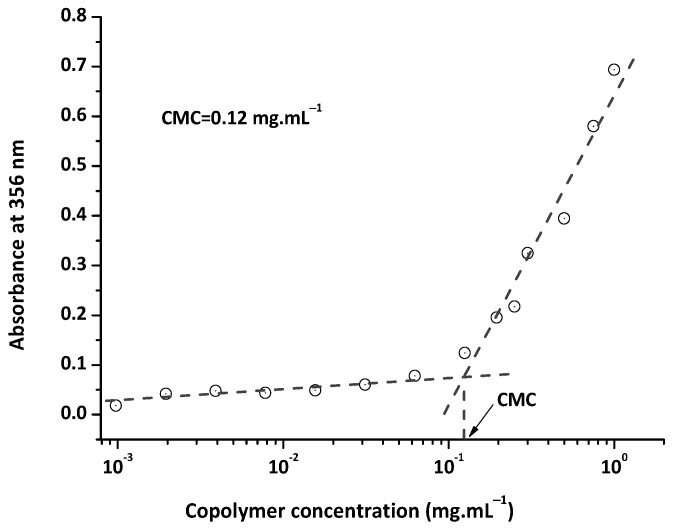
Determination of the CMC of the PG_50_-*b*-PPO_4_-*b*-[P(CyCL)_4_-*co*-(CL)_40_]-*b*-PPO_4_-*b*-PG_50_ copolymer using 1,6-diphenyl-1,3,5-hexatriene absorbance at 356 nm in aqueous media at 25 °C.

The critical micelle concentration of the modified copolymer was determined by dye solubilization. The method employs specific photophysical properties of the hydrophobic dye 1,6-diphenyl-1,3,5-hexatriene: its UV absorbance in water is minimal, whereas it increases substantially in a hydrophobic environment so that the appearance of a characteristic maximum at 356 nm and its sharp increase upon increasing copolymer concentration indicates an abrupt change in the properties of the system— the formation of hydrophobic domains (presumably cores of the micelles). DPH solubilization has frequently been employed for the determination of the CMC of conventional surfactants and amphiphilic polymers [48,61,62,63]. The CMC value was determined from the break of the absorbance intensity vs. copolymer concentration curve as shown in Figure 2. 

The resulting value (0.12 mg·mL^−1^) was slightly larger than that of the reference copolymer PG_45_-*b*-PCL_35_-*b*-PG_45_ (0.10 mg·mL^−1^) [48] and probably reflected the effects of the longer hydrophilic polyglycidol blocks.

At concentrations ca. one order of magnitude higher than the CMC, the copolymer micelles were loaded with CBD by applying different protocols, depicted in Scheme 1—loading during micelle formation (protocol A) and loading in preformed micelles (protocol B). Based on previous studies [51,53] and preliminary concentration-dependent measurements, the drug/copolymer-mass ratio of 1:10 at a copolymer concentration of 1 mg·mL^−1^ was determined to be the most effective and optimal, ensuring the highest encapsulation efficiency and drug loading. Preliminary screening of the size and size distributions, performed by DLS at a single angle (90°), revealed monomodal size distributions and apparent hydrodynamic radii, R_h_^90^, in the 50–60 nm range (Table 2). All particles exhibited low ζ potential (very slightly positive or negative, practically neutral), which is in line with the non-ionic nature of the two copolymers and the drug (Table 2). The simultaneous formation and loading of the micelles (protocol A) yielded higher encapsulation efficiency than protocol B, that is, the loading of the drug into preformed micelles (Table 2). The aggregates of the novel copolymer invariably exhibited higher encapsulation efficiency than those of the referent copolymer (Table 2), which apparently reflected the introduction of cinnamyl-bearing units intended to increase the loading efficiency. Although exhibiting low ζ potential, the micelles of the two copolymers (blank or CBD-loaded) are characterized by enhanced colloidal stability for at least three months. The enhanced colloidal stability is provided by the hydrophilic corona built of polyglycidol chains.

**Figure 3:**
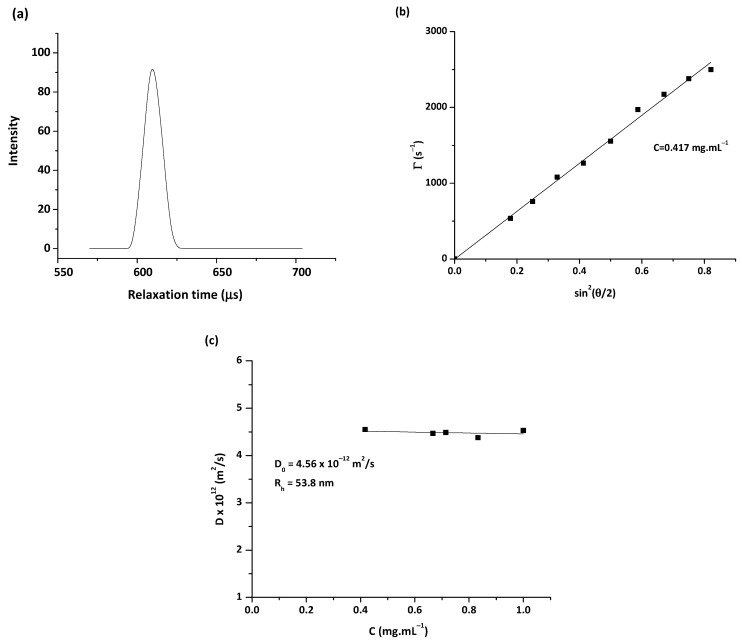
(**a**) Representative relaxation time distribution (τ), measured at an angle of 90° for aqueous solution of the loaded micelles of the novel copolymer at a concentration of 1.0 mg·mL^−1^. (**b**) Relaxation rate (Γ) as a function of sin^2^(θ/2) for the loaded micelles of the novel copolymer at a concentration of 0.417 mg·mL^−1^. (**c**) Concentration dependence of diffusion coefficients for the loaded micelles of the novel copolymer. The lines through the data points in (**b**,**c**) represent the linear fit to the data. Measurements were performed at 25 °C.

Multiangle dynamic and static light scattering was performed to fully characterize the empty micelles of the novel copolymer and the micelles of the novel copolymer loaded by protocol A. In the investigated concentration range (0.417–1.0 mg·mL^−1^), which is slightly above the CMC, the relaxation time distributions were predominantly monomodal, indicating the existence of only one population of particles. Additional modes of low amplitude (mainly fast modes of amplitude below 4%) were occasionally and unsystematically observed at certain angles. A representative time distribution is shown in Figure 3a. More relaxation time distributions and converted therefrom particle size distributions are presented in Figure S4 in the ESI. The diffusion coefficients were determined from the angular dependence of the relaxation rate (inversely proportional to relaxation time), measured at different scattering angles and then plotted against concentration to obtain the diffusion coefficient at zero concentration, D_0_, as shown in Figure 3b and c. D_0_ was used to calculate the hydrodynamic radii, R_h_, of the empty and loaded micelles. The R_h_ values of the empty and CBD-loaded micelles are summarized in Table 3.

**Figure 4:**
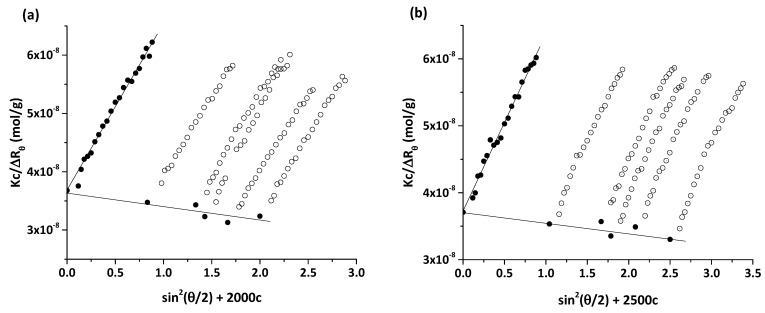
Zimm plots of empty (**a**) and CBD-loaded (**b**) micelles of the novel copolymer in aqueous solution. Open symbols and closed symbols represent experimental points and extrapolated points of zero concentration and zero angle, respectively. Measurements were performed at 25 °C.

Static light scattering was performed to determine the weight-average molar mass (M_w_), radius of gyration (R_g_), and second virial coefficient (A_2_) of the empty and loaded micelles. The static parameters were evaluated by the Zimm plot method. Zimm diagrams of the empty and loaded micelles are presented in Figure 4, whereas the derived parameters are collected in Table 3. 

Evident from the results in Table 3 is that the CBD-loaded micelles are slightly smaller in size than the empty micelles, whereas the molar masses are comparable within the standard deviation of the method. These findings implied the formation of more compact and dense particles and revealed the effect of the hydrophobic drug molecules as nucleation sites on which copolymer self-assembly occurred, bringing about the enhancement of particle density. A realistic assessment of the different compactness levels of the empty and loaded particles is given by the particle density, ρ (Table 3), calculated from the molar mass and hydrodynamic volume data while assuming the spherical morphology of the particles (see ESI for the calculation of the particle density). Apparently, the ρ value of the CBD-loaded micelles was higher than that of the empty micelles. In addition, R_g_/R_h_ attained values close to unity, which was compatible with the structure of both the empty and loaded micelles—relatively dense and compact particles with “hairy” surfaces [64,65]. A_2_ values were very small in magnitude (of the order of 10^−6^ mL·mol/g^2^), which is in accordance with the high molar mass of the micelles, and negative. The negative values of A_2_ normally indicate unfavorable particle–solvent interactions. As the A_2_ values are very small, we may speculate here that there are weak attractive interactions between the micelles, previously observed for self-assembled structures of polyglycidol-based copolymers [48,66,67].

Another parameter that can be extracted from the light scattering data is the loading capacity expressed as the number of CBD molecules loaded in one micelle of the novel and referent copolymer. The light scattering characterization data of the referent copolymer micelles loaded with CBD as well as the calculation of loading capacity are presented in Figure S5 and Table S1 in the ESI. Evident from the values of this quantity was that the micelles of the novel copolymer bore about 32% more CBD molecules than those of the referent copolymer, which undoubtedly revealed the effect of the copolymer design, namely, the introduction of cinnamyl-bearing units in the PCL block of the copolymer. Measurements were also performed at the physiological temperature of 37 °C. As many of the constituent elements of the copolymer micelles did not exhibit- sensitivity to temperature variations in this temperature range, the characterization parameters were practically the same as those at 25 °C and, therefore, not presented. 

Furthermore, data for the enhanced encapsulation efficiency and loading capacity of the modified block copolymer micelles were in excellent agreement with the calculated Flory–Huggins solubilization parameters (χ_sp_) for the two core-forming blocks (Table S2 in the ESI). χ_sp_ for the modified block P(CyCL-*co*-CL) was 0.0039 vs. 0.1195 for PCL, indicating the significantly greater affinity of the former to CBD [68,69,70].

**Figure 5:**
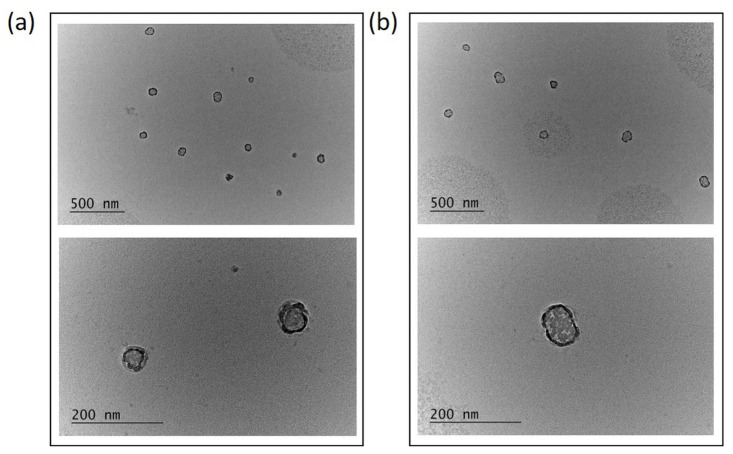
Transmission electron micrographs of blank (**a**) and loaded with CBD (**b**) PG_50_-*b*-PPO_4_-*b*-[P(CyCL)_4_-*co*-(CL)_40_]-*b*-PPO_4_-*b*-PG_50_ micelles. Samples were stained with uranyl acetate.

**Figure 6:**
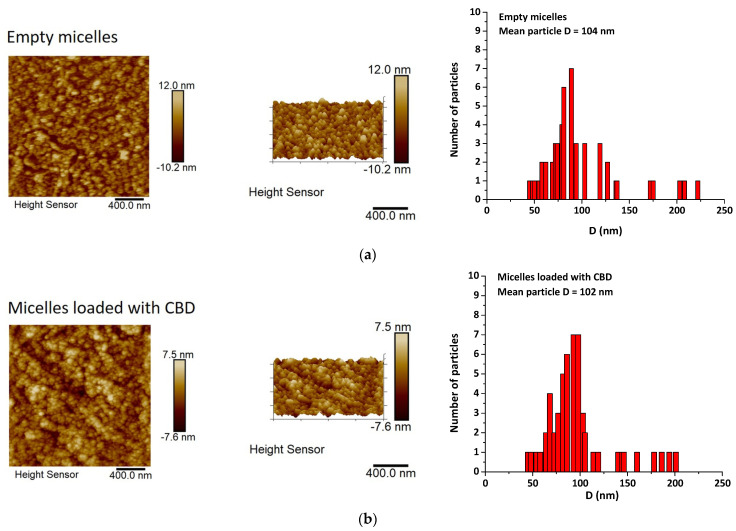
Representative AFM images, particle size (diameter), and size distributions of micelles deposited from their aqueous dispersion. Empty micelles (**a**) and micelles loaded with CBD (**b**) deposited from 1.0 mg·mL^−1^ dispersion.

TEM and AFM were performed to resolve the morphology of the empty and CBD-loaded micelles (Figure 5 and Figure 6). The objects were well-separated with dimensions in a dry state that was consistent with the results from the dynamic light scattering. The spherical morphology is dominant; some irregularities in the sphericity were occasionally observed and could be attributed to dehydration-induced artifacts. We suggested that the increased contrast at the periphery of the micelles was due to a selective interaction between PG corona and the staining agent—uranyl acetate. The loading of the micelles with CBD did not affect their morphology. 

### 3.3. In Vitro Drug Release and Cytotoxicity Assay

**Figure 7:**
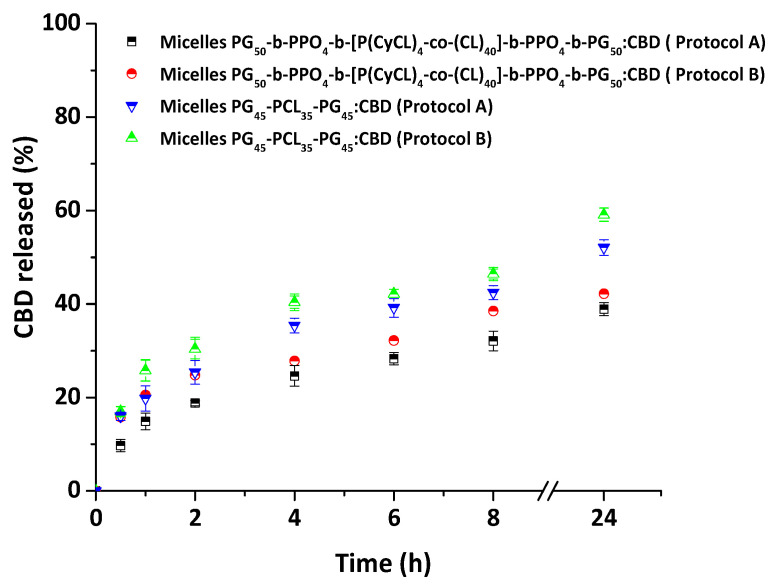
In vitro release of CBD from block copolymer micelles in phosphate buffer (pH = 7.4). The copolymer/CBD weight ratio is 10:1.

CBD release from prepared micelles was investigated by regular dialysis against PBS at 37 °C and the results are presented in Figure 7. Evident from the presented results is that the elaborated nanocarriers are able to release their cargo in a sustained manner for a prolonged period of time. This effect was more pronounced for micelles based on PG_50_-*b*-PPO_4_-*b*-[P(CyCL)_4_-*co*-(CL)_40_]-*b*-PPO_4_-*b*-PG_50_ where less than 45% of the encapsulated CBD was released at the 24th hour, compared to nearly 60% CBD released from the referent PG_45_-*b*-PCL_35_-*b*-PG_45_ micelles. This was probably due to the higher affinity of CBD to the cinnamyl-modified core and hence better solubilizing ability and loading efficiency, and this was in line with other reported studies [71]. The method of preparation of the micelles also had an effect on the release profile of CBD. In both types of micelles, those prepared according to protocol B showed a faster release, which can be explained by the entrapment of CBD in the periphery of the PCL core or even in the core–corona interface (Scheme 1a), rather than in the interior of the core. Indeed, PCL is a crystallizable polymer, and micelles based on this polymer are kinetically frozen structures, the hydrophobic domains of which can hardly be deeply penetrated by the external addition of the drug to preformed micelles. In contrast, in micelles prepared according to protocol A, the dominant location of CBD is likely to be well in the interior of the micellar core (Scheme 1b). It is anticipated that such localization would slow down the diffusion and, hence, the release of the drug [72]. In addition, the physicochemical affinity of CBD with the cinnamyl residues may further contribute to the slower drug release. 

In order to elucidate the release mechanism of CBD from the micelles, the data from the release profiles were fitted by linear regression to several kinetic models: zero and first order Higuchi and Korsmeyer–Peppas (Figures S6 and S7). Data are presented in Table 4. For a more accurate interpretation of the data from the dissolution test, we additionally conducted a non-linear regression analysis [73,74] whereby the drug-release data were fitted to the Korsmeyer–Peppas model using DDSolver—a freely available Excel plug-in software [75]. The results are summarized in Table S3. The obtained correlation coefficient data (Table S3) coincided with the corresponding values from the linear analysis (Table 4), which proved the accuracy of the model. As can be seen from the data, the best correlation was observed with the Korsmeyer–Peppas kinetic model, with n-values found for all tested formulations below 0.45, indicating that the release followed a mechanism of typical Fick diffusion [76]. However, taking into account the low values of the kinetic parameters (release rate constant and half-release time) calculated for the different models (Table 4), it can be assumed that the CBD release mechanism is rather complex and, in addition to the diffusion, the probable redistribution of the released drug in the micelle has an influence due to its strong hydrophobicity.

**Figure 8:**
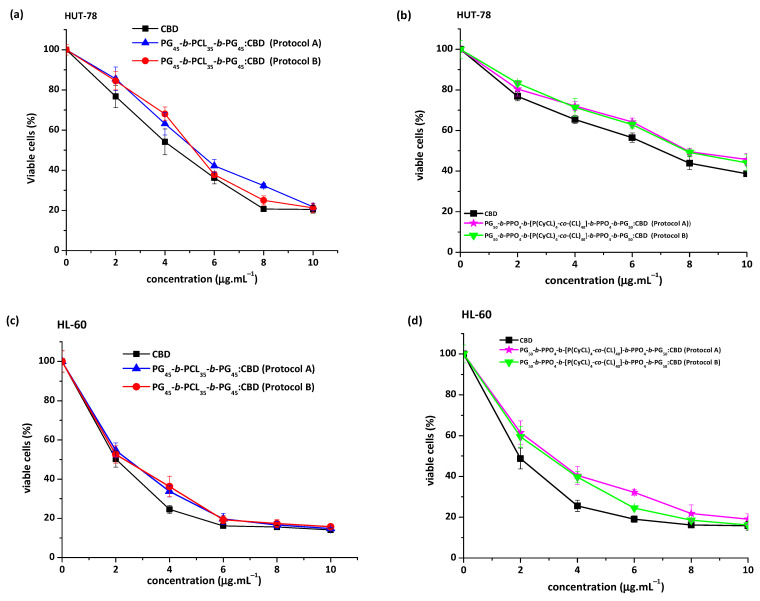
Cytotoxicity of micellar vs. free CBD (as ethanol solution) against HUT-78 (**a**,**b**) and HL-60 (**c**,**d**) human tumor cell lines after 72 h continuous exposure at 37 °C. Loaded polymer micelles were prepared via protocols A and B. Each data point represents the arithmetic mean ± SD of six separate experiments. The copolymer: CBD-mass ratio is 10:1.

A comparative evaluation of the antiproliferative effect of micellar CBD vs. free drug (applied as ethanol solution) against the acute myeloid leukemia-derived HL-60 cell line and Sezary Syndrome HUT-78 was performed. The growth-inhibitory concentration–response curves are shown in Figure 8 and the derived thereof equieffective IC_50_ values are presented in Table 5. As seen from the presented data, the micellar CBD showed slightly lower cytotoxicity as compared to the free drug in both tested tumor lines, thus the concentration–response curves were shifted to the higher concentrations and, respectively, the IC_50_ values were higher as compared to those of non-formulated drug, applied as an ethanol solution. Based on the presented results, it is clear that the incorporation of CBD into micelles has a modulatory effect on its range of antitumor activity, possibly due to changes in the release kinetics. These findings paralleled those of the drug-release study, where slower CBD release was reported for both micellar formulations.

**Figure 9:**
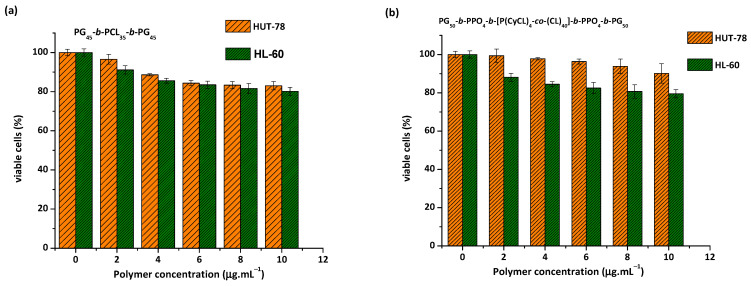
Cytotoxicity of empty micelles of the referent copolymer PG_45_-*b*-PCL_35_-*b*-PG_45_ (**a**) and novel copolymer PG_50_-*b*-PPO_4_-b-[P(CyCL)_4_-*co*-(CL)_40_]-*b*-PPO_4_-*b*-PG_50_ (**b**) against HUT-78 and HL-60 cell lines after 72 h continuous exposure at 37 °C.

To prove that the observed cytotoxicity of micellar CBD is mainly due to the inherent cytotoxicity of CBD and not the carrier, the antiproliferative effect of unloaded micelles was also investigated in the same concentration range as that of the loaded counterparts (Figure 9). The obtained results showed that the used polymeric micelles were devoid of cytotoxic potential. No significant differences to the untreated control were measured.

## 4. Conclusions

Aiming at increasing the encapsulation efficiency and improving the performance of copolymer micelles as delivery systems of CBD, we designed a novel PEO-free PCL-PG copolymer by introducing a small number (an average of 4 out of 44) of monomer units bearing pendant cinnamyl groups in the middle block of PCL. Azide-alkyne click reactions were employed for the attachment of the pendant groups and for the conjugation of flanking polyether blocks to the middle polyester block. Amphiphilic properties were conferred upon, converting the flanking blocks into blocks of linear polyglycidol by removing the protective EEGE groups. In aqueous solution, the copolymer was found to spontaneously self-associate above a certain critical concentration into well-defined spherical micelles, characterized by moderately large size (R_h_ = 59.1 nm) and molar mass (M_w_ = 27.560 × 10^6^ g/mol), and slightly negative (−5.90 mV) ζ potential. The micelles of the novel copolymer exhibited higher encapsulation efficiency towards CBD than those of the referent copolymer, independently from the loading protocols applied. Furthermore, the data revealed the enhancement of the loading capacity, expressed as the number of CBD molecules per micelle, by ca. 1/3 as compared to the referent copolymer micelles. Loading CBD during micelle formation (protocol A) resulted in the formation of more dense and compact particles, which together with embedding CBD molecules into the interior of the micellar cores strongly reduced the initial burst effect and prolonged the drug release from the carriers. The copolymers displayed no sign of toxicity, whereas the cytotoxic activity and antiproliferative effect of CBD loaded in the micelles, against acute myeloid leukemia-derived HL-60 cell line and Sezary Syndrome HUT-78, was retained. The results of this demonstrate the effective design of a novel copolymer and the potential of its micelles as delivery vehicles of CBD. With their ability to significantly enhance the solubility of CBD and its loading efficiency, favorable physicochemical characteristics, appropriate release profiles, and excellent biocompatibility, the micelles of the novel copolymer can further enhance the experimental knowledge and therapeutic potential of CBD in neurological diseases and cancer.

## Figures and Tables

**Table 1:** Composition and molar mass characteristics of the synthesized block copolymers.

Copolymer	M_n_^a^ (g·mol^−1^)	M_w_^b^ (g·mol^−1^)	M_w_/M_n_^b^
PEEGE_45_-*b*-PCL_35_-*b*-PEEGE_45_	17,400	15,300	1.8
PG_45_-*b*-PCL_35_-*b*-PG_45_	11,000	-	-
PEEGE_50_-*b*-PPO_4_-*b*-[P(CyCL)_4_-*co*-(CL)_40_]-*b*-PPO_4_-*b*-PEEGE_50_	20,100	15,000	1.3
PG_50_-*b*-PPO_4_-*b*-[P(CyCL)_4_-*co*-(CL)_40_]-*b*-PPO_4_-*b*-PG_50_	13,200	-	-

^a^—number-averaged molar mass from ^1^H NMR. ^b^—weight-averaged molar mass from SEC; M_w_/M_n_—molar mass distribution from SEC.

**Table 2:** Dynamic and electrophoretic light scattering characterization data and encapsulation efficiencies of the micelles prepared from block copolymers.

Copolymer	R_h_^90^ (nm)			ζ Potential (mV)			EE (%)	
	Blank	Loaded Prot. A	Loaded Prot. B	Blank	Loaded Prot. A	Loaded Prot. B	Loaded Prot. A	Loaded Prot. B
PG_45_-*b*-PCL_35_-*b*-PG_45_	52.0 ± 1.7	56.0 ± 2.7	50.0 ± 3.8	2.94 ± 1.97	−2.61 ± 2.10	2.90 ± 2.40	91.0	82.0
PG_50_-*b*-PPO_4_-*b*-[P(CyCL)_4_-*co*-(CL)_40_]-*b*-PPO_4_-*b*-PG_50_	60.0 ± 1.6	51.0 ± 1.8	57.0 ± 1.4	−5.90 ± 1.23	−6.61 ± 3.10	4.19 ± 2.50	95.0	92.0

**Table 3:** Static and dynamic light scattering characterization data of the empty and CBD-loaded micelles of the novel copolymer. Measurements were performed at 25 °C. The standard deviations in the static and dynamic light scattering parameters are up to 4%.

	R_h_ (nm)	10^−6^ × M_w_ (g·mol^−1^)	R_g_ (nm)	R_g_/R_h_	10^6^ × A_2_ mL·mol/g^2^	ρ ^a^ (mg·mL^−1^)	Loading Capacity ^b^
Empty	59.1	27.560	59.1	1.00	−2.6	52.9	n.a.
CBD-loaded	53.8	26.970	54.2	1.03	−2.8	68.7	8161

^a^—density of the material within the particle. ^b^—expressed as the number of CBD molecules loaded in one copolymer micelle.

**Table 4:** Coefficient of determination (R^2^), release rate constant (K), release half time (t_1/2_), and diffusion exponent (n), after fitting of release profiles to different drug-release kinetic models.

Kinetic Model	Zero Order			First Order			Higuchi		Korsmeyer–Peppas		
Sample	R^2^	K_o_ (mg·mL^−1^)/h	t_1/2_ (h)	R^2^	K_F_ (h^−1^)	t_1/2_ (h)	R^2^	K_H_ (mg·mL^−1^)/h^0.5^	R^2^	K_KP_ (h^n^)	n
PG_50_-b-PPO_4_-b-[P(CyCL)_4_-*co*-(CL)_40_]-b-PPO_4_-b-PG_50_:CBD (protocol A)	0.746	1.148	0.043	0.808	−0.007	99	0.943	7.467	0.978	−0.845	0.366
PG_50_-*b*-PPO_4_-*b*-[P(CyCL)_4_-*co*-(CL)_40_]-*b*-PPO_4_-*b*-PG_50_:CBD (protocol B)	0.782	1.017	0.049	0.707	−0.006	115.5	0.946	6.341	0.970	−0.692	0.261
PG_45_-*b*-PCL_35_-*b*-PG_45_:CBD (protocol A)	0.675	1.381	0.036	0.753	−0.010	69.3	0.926	9.562	0.986	−0.696	0.353
PG_45_-*b*-PCL_35_-*b*-PG_45_:CBD (protocol B)	0.722	1.495	0.033	0.886	−0.016	43.31	0.957	9.955	0.977	−0.615	0.315

**Table 5:** Equieffective concentrations (IC_50_) values in µg·mL^−1^ of free CBD (as ethanol solution) and loaded polymer micelles prepared via protocols A and B towards HL-60 and HUT-78 cell lines.

Sample	IC_50_			
	HL-60		HUT-78	
	Protocol A	Protocol B	Protocol A	Protocol B
PG_45_-*b*-PCL_35_-*b*-PG_45_:CBD	2.33	2.29	5.26	5.17
PG_50_-*b*-PPO_4_-*b*-[P(CyCL)_4_-*co*-(CL)_40_]-*b*-PPO_4_-*b*-PG_50_:CBD	3.00	3.20	8.30	8.00
Pure CBD	2.00		7.00	

## Data Availability

Data is contained within the article.

## Supplementary Materials

The following supporting information can be downloaded at: https://www.mdpi.com/article/10.3390/pharmaceutics15082128/s1, Section S1: Structure and physicochemical properties of cannabidiol (CBD); Section S2: Reaction scheme, molar mass characteristics, and composition of PCL-cinnamyl precursor; Section S3: Monoalkyne-terminated poly(ethoxyethyl glycidyl ether) (tBu-PEEGE_50_-b-PPO_4_-C≡CH); Section S4: Synthesis and characterization data of the referent copolymer. Section S5: ^1^H NMR spectra of the novel PEEGE_50_-b-PPO_4_-b-[P(CyCL)_4_-co-(CL)_40_]-b-PPO_4_-b-PEEGE_50_ and PG_50_-b-PPO_4_-b-[P(CyCL)_4_-co-(CL)_40_]-b-PPO_4_-b-PG_50_ block copolymers; Section S6: Relaxation time distributions and converted therefrom particle size distributions; Section S7: Calculation of density of the material within the particle, ρ; Section S8: Light scattering characterization data of the referent copolymer micelles loaded with CBD; Section S9: Determination of loading capacity (number of CBD molecules per micelle); Section S10: Determination of the Flory–Huggins parameter; Section S11: Release profiles fitting; Scheme S1: Chemical structure of cannabidiol; Scheme S2: Schematic presentation of the synthesis of the bifunctional macroreagent N_3_-[P(CyCL)_4_-co-(CL)_40_]-N_3_; Scheme S3: Schematic presentations of the synthesis of mono-alkyne functional *tBu*-PEEGE50-b-PPO4-C≡CH via esterification of tBu-PEEGE50-b-PPO4-OH with 4-pentynoic acid; Figure S1: SEC chromatogram of tBu-PEEGE50-b-PPO4-OH and tBu-PEEGE50-b-PPO4-C≡CH (RI trace, THF); Figure S2: 1H NMR spectra of tBu-PEEGE50-b-PPO4-OH and tBu-PEEGE50-b-PPO4-C≡CH in CD3OH; Figure S3: 1H NMR spectra of (a) PEEGE50-b-PPO4-b-[P(CyCL)4-co-(CL)40]-b-PPO4-b-PEEGE50 in CDCl3 and (b) PG50-b-PPO4-b-[P(CyCL)4-co-(CL)40]-b-PPO4-b-PG50 in CD3OH; Figure S4: Relaxation time distributions (a,c,e) and the corresponding particle size distributions (b,d,f) from DLS, measured at an angle of 70° and concentration of 0.833 mg/mL (a,b), 90° and 0.714 mg/mL (c,d), and 110° and 0.417 mg/mL (e and f) for aqueous dispersions of the CBD-loaded micelles of the novel copolymer PG50-b-PPO4-b-[P(CyCL)4-co-(CL)40]-b-PPO4-b-PG50; Figure S5: Zimm plot of CBD-loaded micelles of the referent copolymer in aqueous solution. Blue symbols represent experimental points. Red and purple symbols represent extrapolated points of zero concentration and zero angles, respectively. Measurements were performed at 25 °C; Figure S6: Release profiles of CBD from micelles of PG50-b-PPO4-b-[P(CyCL)4-co-(CL)40]-b-PPO4-b-PG50PG45-b-PCL35-b-PG45 (a,b) and PG45-b-PCL35-b-PG45 (c,d), loaded according to protocol A (a,c) and protocol B (b,d) and fitting to the Higuchi kinetic model; Figure S7: Release profiles of CBD from micelles of PG50-b-PPO4-b-[P(CyCL)4-co-(CL)40]-b-PPO4-b-PG50PG45-b-PCL35-b-PG45 (a,b) and PG45-b-PCL35-b-PG45 (c,d), loaded according to protocol A (a,c) and protocol B (b,d) and fitting to the Korsmeyer–Peppas kinetic model; Table S1: Static light scattering characterization data of the CBD-loaded micelles of the referent copolymer PG45-b-PCL35-b-PG45. Measurements were performed at 25 °C. The standard deviations in the static light scattering parameters are up to 4%. a—expressed as the number of CBD molecules loaded in one copolymer micelle; Table S2: Calculated values for solubility parameters (δ), drug–polymer compatibility (χsp) for PCL- and P-containing copolymers; Table S3: Coefficient of determination (R2), release rate constant (K) and diffusion exponent (n), after fitting of release profiles to Korsmeyer–Peppas kinetic model by non-linear regression analysis, using freely available Excel plug-in software DDSolver. References [51,52,54,55,68,69,70,75] are cited in the supplementary materials.

## References

[1] Liechty W.B., Kryscio D.R., Slaughter B.V., Peppas N.A. (2010). Polymers for drug delivery systems. Annu. Rev. Chem. Biomol. Eng..

[2] Nicolas J. (2016). Drug-Initiated Synthesis of Polymer Prodrugs: Combining Simplicity and Efficacy in Drug Delivery. Chem. Mater..

[3] Liu S., Maheshwari R., Kiick K.L. (2009). Polymer-Based Therapeutics. Macromolecules.

[4] Zhou W., Li C., Wang Z., Liu J. (2016). Factors affecting the stability of drug-loaded polymeric micelles and strategies for improvement. J. Nanoparticle Res..

[5] Figueiras A., Domingues C., Jarak I., Santos A.I., Parra A., Pais A., Alvarez-Lorenzo C., Concheiro A., Kabanov A., Cabral H. (2022). New Advances in Biomedical Application of Polymeric Micelles. Pharmaceutics.

[6] Ghosh B., Biswas S. (2021). Polymeric micelles in cancer therapy: State of the art. J Control. Release.

[7] Chen L.G., Strasburg S.H., Bermudez H. (2016). Micelle co-assembly in surfactant/ionic liquid mixtures. J. Colloid Interface Sci..

[8] Hanafy N.A.N., El-Kemary M., Leporatti S. (2018). Micelles Structure Development as a Strategy to Improve Smart Cancer Therapy. Cancers.

[9] Danson S., Ferry D., Alakhov V., Margison J., Kerr D., Jowle D., Brampton M., Halbert G., Ranson M. (2004). Phase I dose escalation and pharmacokinetic study of pluronic polymer-bound doxorubicin (SP1049C) in patients with advanced cancer. Br. J. Cancer.

[10] Lukyanov A.N., Torchilin V.P. (2004). Micelles from lipid derivatives of water-soluble polymers as delivery systems for poorly soluble drugs. Adv. Drug Deliv. Rev..

[11] Deng W., Chen J., Kulkarnia A., Thompson D.H. (2012). Poly(ethylene glycol)-poly(vinyl alcohol)-adamantanate: Synthesis and stimuli-responsive micelle properties. Soft Matter.

[12] Foster J.C., Akar I., Grocott M.C., Pearce A.K., Mathers R.T., O’Reilly R.K. (2020). 100th Anniversary of Macromolecular Science Viewpoint: The Role of Hydrophobicity in Polymer Phenomena. ACS Macro Lett..

[13] Chen S., Guo C., Hu G.H., Wang J., Ma J.H., Liang X.F., Zheng L., Liu H.Z. (2006). Effect of hydrophobicity inside PEO-PPO-PEO block copolymer micelles on the stabilization of gold nanoparticles: Experiments. Langmuir.

[14] Li G., Zhao M., Xu F., Yang B., Li X., Meng X., Teng L., Sun F., Li Y. (2020). Synthesis and Biological Application of Polylactic Acid. Molecules.

[15] Van Butsele K., Cajot S., Van Vlierberghe S., Dubruel P., Passirani C., Benoit J.-P., Jérôme R., Jérôme C. (2009). pH-Responsive Flower-Type Micelles Formed by a Biotinylated Poly(2-vinylpyridine)-block-poly(ethylene oxide)-block-poly(ε-caprolactone) Triblock Copolymer. Adv. Funct. Mater..

[16] Liu S.Q., Tong Y.W., Yang Y.Y. (2005). Incorporation and in vitro release of doxorubicin in thermally sensitive micelles made from poly(N-isopropylacrylamide-co-N,N-dimethylacrylamide)-b-poly(d,l-lactide-co-glycolide) with varying compositions. Biomaterials.

[17] Palao-Suay R., Gómez-Mascaraque L.G., Aguilar M.R., Vázquez-Lasa B., San Román J. (2016). Self-assembling polymer systems for advanced treatment of cancer and inflammation. Prog. Polym. Sci..

[18] Woodruff M.A., Werner Hutmacher D. (2010). The return of a forgotten polymer—Polycaprolactone in the 21st century. Prog. Polym. Sci..

[19] Corrigan N., Manahan R., Lew Z.T., Jonathan Y., Xu J., Boyer C. (2018). Copolymers with Controlled Molecular Weight Distributions and Compositional Gradients through Flow Polymerization. Macromolecules.

[20] Iskin B., Yilmaz G., Yagci Y. (2011). ABC type miktoarm star copolymers through combination of controlled polymerization techniques with thiol-ene and azide-alkyne click reactions. J. Polym. Sci. Part A Polym. Chem..

[21] Atanasova M.D., Grancharov G., Petrov P.D. (2021). Poly(ethylene oxide)-block-poly(α-cinnamyl-ε-caprolactone-co-ε-caprolactone) diblock copolymer nanocarriers for enhanced solubilization of caffeic acid phenethyl ester. J. Polym. Sci..

[22] Kalinova R., Yordanov Y., Tzankov B., Tzankova V., Yoncheva K., Dimitrov I. (2020). Cinnamyl modified polymer micelles as efficient carriers of caffeic acid phenethyl ester. React. Funct. Polym..

[23] Liu S., Kobayashi S., Nishimura S., Ueda T., Tanaka M. (2021). Effect of pendant groups on the blood compatibility and hydration states of poly(2-oxazoline)s. J. Polym. Sci..

[24] Garg S.M., Vakili M.R., Lavasanifar A. (2015). Polymeric micelles based on poly(ethylene oxide) and α-carbon substituted poly(ɛ-caprolactone): An in vitro study on the effect of core forming block on polymeric micellar stability, biocompatibility, and immunogenicity. Colloids Surf. B Biointerfaces.

[25] Son I., Lee Y., Baek J., Park M., Han D., Min S.K., Lee D., Kim B.-S. (2021). pH-Responsive Amphiphilic Polyether Micelles with Superior Stability for Smart Drug Delivery. Biomacromolecules.

[26] Grancharov G., Gancheva V., Kyulavska M., Momekova D., Momekov G., Petrov P. (2016). Functional multilayered polymeric nanocarriers for delivery of mitochondrial targeted anticancer drug curcumin. Polymer.

[27] Hu X., Jing X. (2009). Biodegradable amphiphilic polymer–drug conjugate micelles. Expert Opin. Drug Deliv..

[28] Akter S., Clem B.F., Lee H.J., Chesney J., Bae Y. (2011). Block Copolymer Micelles for Controlled Delivery of Glycolytic Enzyme Inhibitors. Pharm. Res..

[29] Zhao Y. (2009). Photocontrollable block copolymer micelles: What can we control?. J. Mater. Chem..

[30] Lee H.J., Bae Y. (2013). Brushed Block Copolymer Micelles with pH-Sensitive Pendant Groups for Controlled Drug Delivery. Pharm. Res..

[31] Yin W., Wang Y., Xiao Y., Mao A., Lang M. (2022). Phenylboronic acid conjugated mPEG-b-PCL micelles as DOX carriers for enhanced drug encapsulation and controlled drug release. Eur. Polym. J..

[32] Shao H., Zhang M., He J., Ni P. (2012). Synthesis and characterization of amphiphilic poly(ɛ-caprolactone)-b-polyphosphoester diblock copolymers bearing multifunctional pendant groups. Polymer.

[33] Lee C.C., Su Y.C., Ko T.P., Lin L.L., Yang C.Y., Chang S.S., Roffler S.R., Wang A.H. (2020). Structural basis of polyethylene glycol recognition by antibody. J. Biomed. Sci..

[34] Suk J.S., Xu Q., Kim N., Hanes J., Ensign L.M. (2016). PEGylation as a strategy for improving nanoparticle-based drug and gene delivery. Adv. Drug Deliv. Rev..

[35] Hoang Thi T.T., Pilkington E.H., Nguyen D.H., Lee J.S., Park K.D., Truong N.P. (2020). The Importance of Poly(ethylene glycol) Alternatives for Overcoming PEG Immunogenicity in Drug Delivery and Bioconjugation. Polymers.

[36] Shi D., Beasock D., Fessler A., Szebeni J., Ljubimova J.Y., Afonin K.A., Dobrovolskaia M.A. (2022). To PEGylate or not to PEGylate: Immunological properties of nanomedicine’s most popular component, polyethylene glycol and its alternatives. Adv. Drug. Deliv. Rev..

[37] Fang Y., Xue J., Gao S., Lu A., Yang D., Jiang H., He Y., Shi K. (2017). Cleavable PEGylation: A strategy for overcoming the “PEG dilemma” in efficient drug delivery. Drug Deliv..

[38] Lila A.S.A., Nawata K., Shimizu T., Ishida T., Kiwada H. (2013). Use of polyglycerol (PG), instead of polyethylene glycol (PEG), prevents induction of the accelerated blood clearance phenomenon against long-circulating liposomes upon repeated administration. Int. J. Pharm..

[39] Tully M., Dimde M., Weise C., Pouyan P., Licha K., Schirner M., Haag R. (2021). Polyglycerol for Half-Life Extension of Proteins—Alternative to PEGylation?. Biomacromolecules.

[40] Maruyama K., Okuizumi S., Ishida O., Yamauchi H., Kikuchi H., Iwatsuru M. (1994). Phosphatidyl polyglycerols prolong liposome circulation in vivo. Int. J. Pharm..

[41] Dworak A., Walach W., Trzebicka B. (1995). Cationic polymerization of glycidol. Polymer structure and polymerization mechanism. Macromol. Chem. Phys..

[42] Thomas A., Müller S.S., Frey H. (2014). Beyond Poly(ethylene glycol): Linear Polyglycerol as a Multifunctional Polyether for Biomedical and Pharmaceutical Applications. Biomacromolecules.

[43] Kainthan R.K., Janzen J., Levin E., Devine D.V., Brooks D.E. (2006). Biocompatibility Testing of Branched and Linear Polyglycidol. Biomacromolecules.

[44] Taton D., Le Borgne A., Sepulchre M., Spassky N. (1994). Synthesis of chiral and racemic functional polymers from glycidol and thioglycidol. Macromol. Chem. Phys..

[45] Dworak A., Baran G., Trzebicka B., Wałach W. (1999). Polyglycidolblock-poly(ethylene oxide)-block-polyglycidol: Synthesis and swelling properties. React. Funct. Polym..

[46] Pouyan P., Cherri M., Haag R. (2022). Polyglycerols as Multi-Functional Platforms: Synthesis and Biomedical Applications. Polymers.

[47] Erberich M., Keul H., Möller M. (2007). Polyglycidols with two orthogonal protective groups: Preparation, selective deprotection, and functionalization. Macromolecules.

[48] Toncheva-Moncheva N., Bakardzhiev P., Rangelov S., Trzebicka B., Forys A., Petrov P.D. (2019). Linear Amphiphilic Polyglycidol/Poly(ε-caprolactone) Block Copolymers Prepared via “Click” Chemistry-Based Concept. Macromolecules.

[49] Bakardzhiev P., Toncheva-Moncheva N., Mladenova K., Petrova S., Videv P., Moskova-Doumanova V., Topouzova-Hristova T., Doumanov J., Rangelov S. (2020). Assembly of amphiphilic nucleic acid-polymer conjugates into complex superaggregates: Preparation, properties, and in vitro performance. Eur. Polym. J..

[50] Maroon J., Bost J. (2018). Review of the neurological benefits of phytocannabinoids. Surg. Neurol. Int..

[51] Vlad R.A., Hancu G., Ciurba A., Antonoaea P., Rédai E.M., Todoran N., Silasi O., Muntean D.L. (2020). Cannabidiol—Therapeutic and legal aspects. Pharmazie.

[52] Williams C.M., Stephens G.J. (2020). Development of cannabidiol as a treatment for severe childhood epilepsies. Br. J. Pharmacol..

[53] Grancharov G., Atanasova M.D., Aluani D., Yoncheva K., Tzankova V., Trusheva B., Forys A., Trzebicka B., Petrov P.D. (2020). Functional block copolymers bearing pendant cinnamyl groups for enhanced solubilization of caffeic acid phenethyl ester. Polym. J..

[54] Namboodiri V.V., Varma R.S. (2002). Solvent-free tetrahydropyranylation (THP) of alcohols and phenols and their regeneration by catalytic aluminum chloride hexahydrate. Tetrahedron Lett..

[55] Dimitrov P., Rangelov S., Dworak A., Haraguchi N., Hirao A., Tsvetanov C.B. (2004). Triblock and radial star-block copolymers comprised of poly(ethoxyethyl glycidyl ether), polyglycidol, poly(propylene oxide) and polystyrene obtained by anionic polymerization initiated by Cs initiators. Macromol. Symp..

[56] Harris J., Roos C., Djalali R., Rheingans O., Maskos M., Schmidt M. (1999). Application of the negative staining technique to both aqueous and organic solvent solutions of polymer particles. Micron.

[57] Provencher S.W. (1979). Inverse Problems in Polymer Characterization: Direct Analysis of Polydispersity with Photon Correlation Spectroscopy. Macromol. Chem..

[58] D’Souza S.S., DeLuca P.P. (2006). Methods to Assess in Vitro Drug Release from Injectable Polymeric Particulate Systems. Pharm. Res..

[59] Mosmann T. (1983). Rapid colorimetric assay for cellular growth and survival: Application to proliferation and cytotoxicity assays. J. Immunol. Methods.

[60] Konstantinov S., Eibl H., Berger M. (1999). BCR-ABL influences the antileukaemic efficacy of alkylphosphocholines. Br. J. Haematol..

[61] Alexandridis P., Holzwarth J.F., Hatton T.A. (1994). Micellization of Poly(ethylene oxide)-Poly(propylene oxide)-Poly(ethylene oxide) Triblock Copolymers in Aqueous Solutions: Thermodynamics of Copolymer Association. Macromolecules.

[62] Scherlund M., Brodin A., Malmsten M. (2000). Micellization and gelation in block copolymer systems containing local anesthetics. Int. J. Pharm..

[63] Halacheva S., Rangelov S., Tsvetanov C. (2008). Synthesis of Polyglycidol-Based Analogues to Pluronic L121–F127 Copolymers. Self-Assembly, Thermodynamics, Turbidimetric and Rheological Studies. Macromolecules.

[64] Burchard W. (1983). Static and Dynamic Light Scattering from Branched Polymers and Biopolymers. In Light Scattering from Polymers. Adv. Polym. Sci..

[65] Burchard W., Brown W. (1996). Light Scattering: Principles and Development.

[66] Rangelov S., Almgren M., Halacheva S., Tsvetanov C. (2007). Polyglycidol-Based Analogues to Pluronic^®^ Copolymers. Light Scattering and Cryogenic Transmission Electron Microscopy Studies. J. Phys. Chem. C.

[67] Rangelov S., Halacheva S., Garamus V., Almgren M. (2008). Structural Polymorphism Exhibited by Polyglycidol-Based Analogues to Pluronic Copolymers in Aqueous Solution. Macromolecules.

[68] Flory P.J. (1953). Principle of Polymer Chemistry.

[69] Fedors R.F. (1974). A method for estimating both the solubility parameters and molar volumes of liquids. J. Polym. Eng. Sci..

[70] Brandrup J., Immergut E.H., Grulke E.A. (1999). Polymer Handbook.

[71] Gupta A., Costa A.P., Xu X., Lee S.-L., Cruz C.N., Bao Q., Burgess D.J. (2020). Formulation and characterization of curcumin loaded polymeric micelles produced via continuous processing. Int. J. Pharm..

[72] Bromberg L. (2008). Polymeric micelles in oral chemotherapy. J. Control. Release.

[73] Tonk S., Rápó E. (2022). Linear and Nonlinear Regression Analysis for the Adsorption of Remazol Dye by Romanian Brewery Waste By-Product, *Saccharomyces cerevisiae*. Int. J. Mol. Sci..

[74] Wu I.Y., Bala S., Škalko-Basnet N., di Cagno M.P. (2019). Interpreting non-linear drug diffusion data: Utilizing Korsmeyer-Peppas model to study drug release from liposomes. Eur. J. Pharm. Sci..

[75] Zhang Y., Huo M., Zhou J., Zou A., Li W., Yao C., Xie S. (2010). DDSolver: An add-in program for modeling and comparison of drug dissolution profiles. AAPS J..

[76] Tao L., Chan J., Uhrich K.E. (2016). Drug loading and release kinetics in polymeric micelles: Comparing dynamic versus unimolecular sugar-based micelles for controlled release. J. Bioact. Compat. Polym..

