# Supplementary material for: Cinnamyl-Modified Polyglycidol/Poly(ε-Caprolactone) Block Copolymer Nanocarriers for Enhanced Encapsulation and Prolonged Release of Cannabidiol
Source: Pharmaceutics. 2023 Aug 13;15(8):2128. doi: 10.3390/pharmaceutics15082128 (PMC10459144; doi:10.3390/pharmaceutics15082128)
Supplement: Supplementary file 1 [file pharmaceutics-15-02128-s001.zip › pharmaceutics-2527219-supplementary.pdf]

pharmaceutics

Article

Cinnamyl-Modified Polyglycidol/Poly(ε-Caprolactone) Block Copolymer Nanocarriers for Enhanced Encapsulation and Prolonged Release of Cannabidiol

Natalia Toncheva-Moncheva [1,*], Erik Dimitrov [1], Georgi Grancharov [1], Denitsa Momekova [2], Petar Petrov [1] and Stanislav Rangelov [1,*]

[1] Institute of Polymers, Bulgarian Academy of Sciences, "Akad. G. Bonchev" Street., bl. 103A, 1113 Sofia, Bulgaria; e_dimitrov@polymer.bas.bg (E.D.); granchar@polymer.bas.bg (G.G.); ppetrov@polymer.bas.bg (P.P.)
[2] Department of Pharmaceutical Technology and Biopharmaceutics, Faculty of Pharmacy, Medical University-Sofia, 2 Dunav Street, 1000 Sofia, Bulgaria; dmomekova@pharmfac.mu-sofia.bg
* Correspondence: ntoncheva@polymer.bas.bg (N.T.-M.); rangelov@polymer.bas.bg (S.R.)

S1. Structure and physicochemical properties of cannabidiol (CBD)

CBD is a phytocannabinoid present in cannabis plants, along with tetrahydrocannabinol and more than 113 other substances belonging to a group of compounds called cannabinoids Scheme S1 [1]. It is a white crystalline powder insoluble in water with molar mass of 314.5 g.mol^{-1} and T_m = 66 °C. CBD is highly efficient for treatment of seizures associated with Lennox-Gastaut syndrome and Dravet syndrome and it is approved by the Food and Drug Administration (FDA) for the for biomedical applications in humans [2].

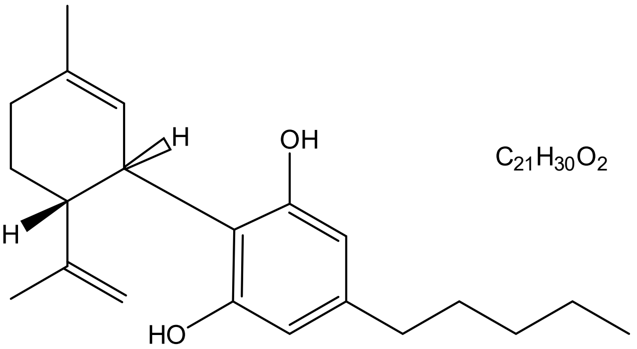

Scheme S1. Chemical structure of cannabidiol.

S2. Reaction scheme, molar mass characteristics, and composition of PCL-cinnamyl precursor

The bifunctional macroreagent N_3-[P(CyCL)$_4$-co-(CL)$_{40}$]-N_3 was obtained by a synthetic procedure involving synthesis of cinnamyl containing PCL diol and functionalization with terminal azide groups. The reaction pathway is presented in Scheme S2.

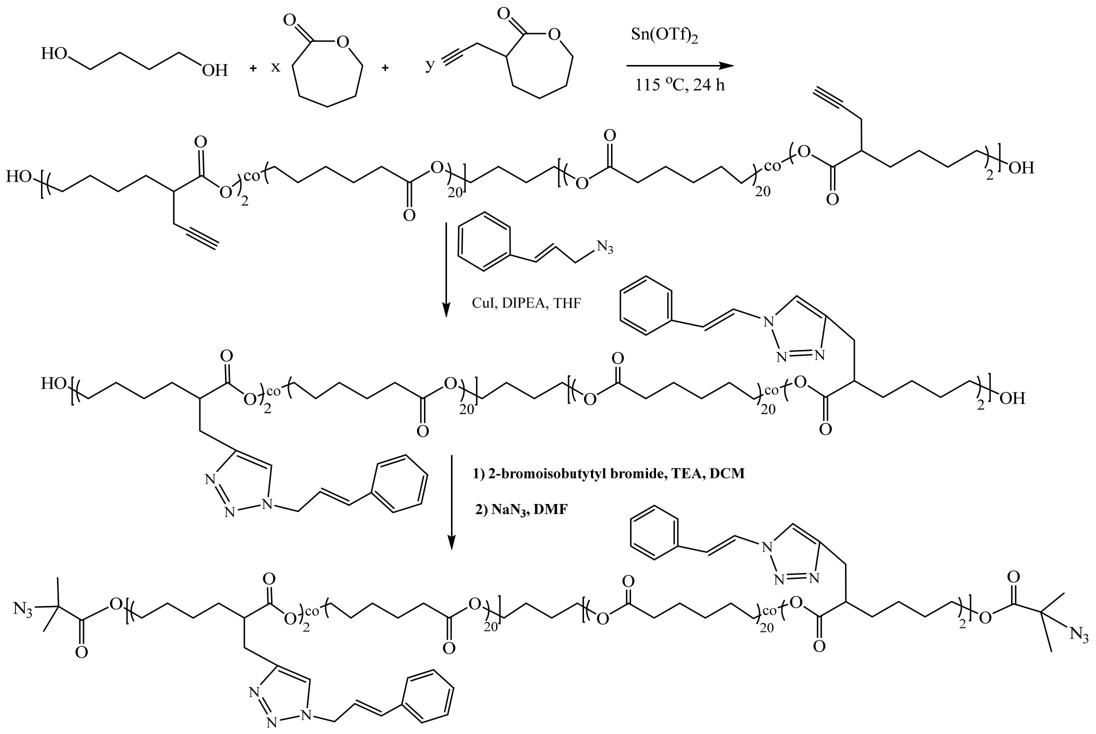

Scheme S2. Schematic presentation of the synthesis of the bifunctional macroreagent N_3-[P(CyCL)$_4$-co-(CL)$_{40}$]-N_3.

The synthetic and characterization details are described below.

Synthesis of α,ω-dihydroxy poly(α-propargyl-ε-caprolactone-co-ε-caprolactone). ε-Caprolactone (2.4 g, 21.0 mmol, 80 eq), α-propargyl-ε-caprolactone (0.8 g, 5.26 mmol, 20 eq), butanediol (23.3 µL, 0.263 mmol, 1 eq) and toluene (8 mL) were placed in a 50 ml two-neck round bottom flask under inert atmosphere. The solution was degassed by argon flow for 20 min and Sn(OTf)$_2$ (55.0 mg, 0.132 mol, 0.5 eq) was added, followed by further degassing for 20 min. The reaction mixture was stirred at room temperature for 48 h and poured into cold isopropanol. The obtained polymer was filtered and thoroughly dried. Yield 64 %, The copolymer contains 8.8 % propargyl units determined by ^{1}H NMR.

$M_{n(1H\ NMR)}$ = 4930 g.mol^{-1}, $M_{n(SEC)}$ = 4100 g.mol^{-1}, M_w/M_n = 1.13.

^{1}H NMR (600 MHz, CDCl$_3$) δ (ppm)= 3.90-4.20 (m, 84H, -C*H*$_2$O-), 3.64 (t, 4H, -C*H*$_2$O-), 2.53-2.58 (m, 4H, COC*H*CH$_2$), 2.45-2.48 (m, 4H, COCHC*H*$_2$), 2.38-2.42 (m, 4H, COCHC*H*$_2$), 2.21-2.35 (m, 80H, COC*H*$_2$), 2.01 (s, 4H, C≡CH), 1.50-1.80 (m, 168H, CH$_2$-C*H*$_2$-C*H*$_2$-CH$_2$-O-), 1.30-1.50 (m, 84H, COCH$_2$C*H*$_2$), 1.17-1.27 (m, 4H, -C*H*$_2$C*H*$_2$-).

Functionalization of α,ω-dihydroxy poly(α-propargyl-ε-caprolactone-co-ε-caprolactone) to α,ω-dihydroxy poly(α-cinnamyl-ε-caprolactone-co-ε-caprolactone) via CuAAC. The copolymer with grafted 8.8 % propargyl units (1.0 g, M_w=4930, 0.203 mmol, i.e. 0.75 mmol, 1 eq of pendant functional groups), CuI (571.4 mg, 3.00 mmol, 4 eq), CAPE-azide (238.8 mg, 1.50 mmol, 2 eq) were placed in a 50 ml two-neck round bottom flask

under inert atmosphere and degassed by 3 vacuum-nitrogen cycles. After that about 5 ml of THF was added and the system was bubbled for approx. 20 min with argon. Then, degassed N,N-diisopropylethylamine (DIPEA) (387.7 mg, 3.00 mmol, 4 eq) was added and the system was bubbled additionally with argon for 20 min. The flask was stirred for 24 h at 35°C. Finally, 50 ml of THF were added and the reaction mixture was passed through a neutral aluminum oxide plug to remove the cupper salts. The mixture was then dissolved in a minimal amount of THF and precipitated into cold isopropanol. The copolymer contains 8.8 % cinnamyl units determined by ^{1}H NMR.

$M_{n(1H\ NMR)}$ = 4930; $M_{n(SEC)}$ = 4100 g.mol^{-1}, M_w/M_n = 1.13.

^{1}H NMR (600MHz, $CDCl_3$) δ (ppm)= 7.15-7.40 (br m, 24H, Ar*H*), 3.90-4.20 (m, 84H, -C*H*$_2$O-), 3.66 (t, 4H, -C*H*$_2$O-), 2.18-2.40 (m, 84H, COC*H*$_2$), 1.50-1.80 (m, 168H, CH_2-C*H*$_2$-C*H*$_2$-CH_2-O-), 1.30-1.50 (m, 84H, $COCH_2$C*H*$_2$), 1.22-1.27 (m, 4H, -C*H*$_2$C*H*$_2$-).

Functionalization of α,ω-dihydroxy poly(α-cinnamyl-ε-caprolactone-co-ε-caprolactone) to α,ω-dibromo poly(α-cinnamyl-ε-caprolactone-co-ε-caprolactone). The copolymer α,ω-dihydroxy (α-cinnamyl-ε-caprolactone-co-ε-caprolactone) (0.5 g, 1 eq, 0.101 mmol, $M_{n(1H\ NMR)}$ =4930 g.mol^{-1}) was dried for 3 h under high vacuum and dissolved in 8 ml of dry toluene in a 50 ml two-neck round bottom flask at room temperature under inert atmosphere. Triethylamine (Et_3N) (40.9 mg, 0.404 mmol, 4 eq,) was added first, followed by 2-bromoisobutiryl bromide (i-BuBr) (92.9 mg, 0.404 mmol, 4 eq,) that was added dropwise. The reaction was carried out for 48 h at room temperature and the product was filtered from HBr:Et_3N salt by blue-stripe paper filter. Further, it was stirred on an activated carbon overnight and filtered again, concentrated by a rotary evaporator, precipitated in 50 ml cold isopropanol, and finally the product was dried thoroughly at 35 °C in the vacuum oven. The copolymer contains 8.8 % cinnamyl groups determined by ^{1}H NMR.

$M_{n\ (1H\ NMR)}$ = 4930 g.mol^{-1}, $M_{n(SEC)}$ = 4100 g.mol^{-1}, M_w/M_n = 1.13.

^{1}H NMR (600MHz, $CDCl_3$): δ (ppm)= 7.15-7.45 (br m, 24H, Ar*H*), 4.17 (t, 4H, -C*H*$_2$O-), 3.80-4.15 (m, 84H, -C*H*$_2$O-), 2.18-2.40 (m, 84H, COC*H*$_2$), 1.92 ppm (s, 12H, -C-C*H*$_3$), 1.50-1.75 (m, 168H, CH_2-C*H*$_2$-C*H*$_2$-CH_2-O-), 1.30-1.45 (m, 84H, $COCH_2$C*H*$_2$), 1.22-1.27 (m, 4H, -C*H*$_2$C*H*$_2$-).

Functionalization of α,ω-dibromo poly(α-cinnamyl-ε-caprolactone-co-ε-caprolactone) to α,ω-diazido poly(α-cinnamyl-ε-caprolactone-co-ε-caprolactone). The copolymer α,ω-dibromo(α-cinnamyl-ε-caprolactone-co-ε-caprolactone) (0.3 g, 0.061 mmol, 1 eq, $Mn_{(1H\ NMR)}$ =4930) was dried for 3 h under high vacuum and dissolved in 3 ml of anhydrous DMF in a 50 ml two-neck round bottom flask at room temperature under inert atmosphere. NaN_3 (79.3 mg, 1.22 mmol, 20 eq) was added, temperature was increased and reaction mixture was

stirred at 50 °C for 48 h. The solution was concentrated under reduced pressure by a rotary evaporator, diluted with 40 mL of CH_2Cl_2, washed with 50 mL of water and 2x50 mL portions of brine. All aqueous layers were extracted further with 40 mL of CH_2Cl_2, the combined CH_2Cl_2 solution was dried on $MgSO_4$, and evaporated under reduced pressure. The product was redissolved in 3 mL of CH_2Cl_2 and precipitated in 30 mL of cold isopropanol. The product was further filtered and washed well with isopropanol to give a macroreagent carrying two azide end-groups. The copolymer contains 8.8 % cinnamyl groups determined by ^{1}H NMR.

$M_{n(1H\ NMR)}$ = 4930 g.mol^{-1}, $M_{n(SEC)}$ = 4800 g.mol^{-1}, M_w/M_n = 1.35.

^{1}H NMR (600MHz, $CDCl_3$): δ (ppm)= 7.20-7.45 (br m, 24H, Ar*H*), 4.17 (t, 4H, -C*H*$_2$O-), 3.85-4.15 (m, 84H, -C*H*$_2$O-), 2.10-2.40 (m, 84H, COC*H*$_2$), 1.50-1.80 (m, 168H, CH_2-C*H*$_2$-C*H*$_2$-CH_2-O-), 1.46 (s, 12H, -C-C*H*$_3$), 1.35-1.42 (m, 84H, $COCH_2$C*H*$_2$), 1.30-1.35 (m, 4H, -C*H*$_2$C*H*$_2$-) (Figure XXX SI). FTIR - new band with small intensity typical for absorption of N_3 group (2110 cm^{-1}) was detected.

S3. Monoalkyne-terminated poly(ethoxyethyl glycidyl ether) (tBu-PEEGE$_{50}$-b-PPO$_4$-C≡CH)

tBu-PEEGE$_{50}$-b-PPO$_4$-C≡CH was synthesized by ring-opening anionic polymerization of ethoxyethyl glycidyl ether (EEGE) and functionalization via introduction of an alkyne end group. The reaction pathway is presented in Scheme S3.

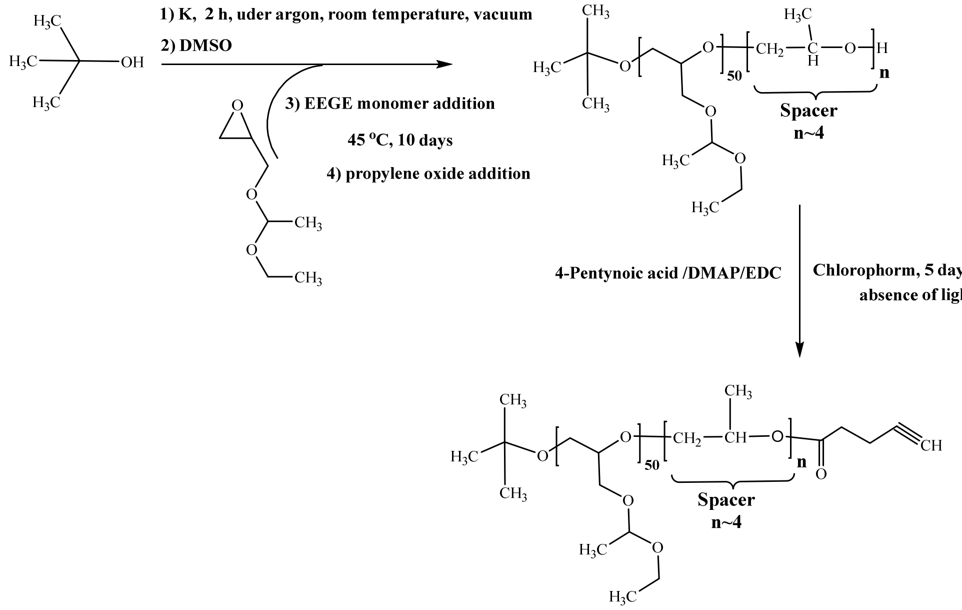

Scheme S3. Schematic presentations of the synthesis of mono-alkyne functional *tBu-PEEGE$_{50}$-b-PPO$_4$-C≡CH* via esterification of *tBu-PEEGE$_{50}$-b-PPO$_4$-OH* with 4-pentynoic acid.

The synthetic and characterization details are described below.

40 mg (1.03 mmol, 1 eq) of metallic potassium was dissolved in 3 ml of absolute tert-butanol into a 50 mL two-neck round-bottom flask under argon atmosphere. The reaction mixture was frozen with liquid nitrogen

and the unreacted alcohol was removed under vacuum. Next, 32 ml of anhydrous DMSO was added and the mixture was magnetically stirred at room temperature for 10 min. Finally, the monomer EEGE (8.3 g, 57 mmol, 55 eq) was added and the polymerization was conducted at 45°C for 10 days under inert atmosphere. Then, the temperature was adjusted at 40 °C, and propylene oxide (0.5 ml, 0.43 g, 7.43 mmol, 7.2 eq) was added to the reaction medium via a Hamilton syringe. After additional 5 days the polymerization was terminated with distilled water. The reaction mixture was extracted 3 times with 50 ml portions of hexane, the combined extracts were dried with $MgSO_4$, filtered and the solvent was evaporated.

Yield: 7.92 g (90%). $M_{n(SEC)}$=6700 g.mol^{-1}, M_w/M_n=1.1 (Figure S1). The degrees of polymerization of PEEGE (= 50) and PPO (= 4) were determined from the ^{1}H NMR spectra (Figure S2).

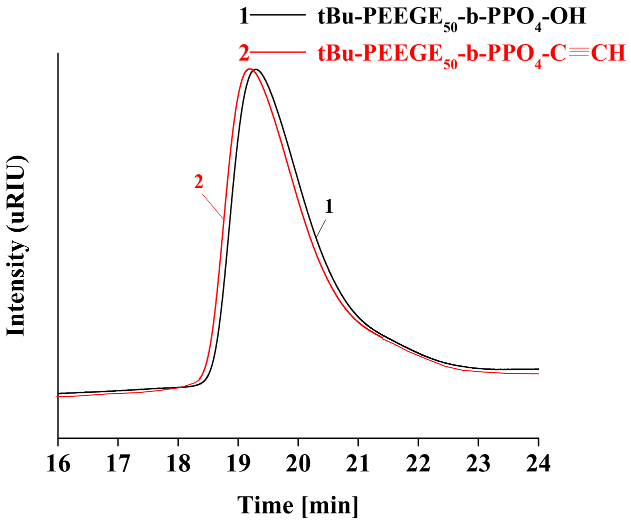

Figure S1. SEC chromatogram of tBu-PEEGE$_{50}$-b-PPO$_4$-OH and tBu-PEEGE$_{50}$-b-PPO$_4$-C≡CH (RI trace, THF).

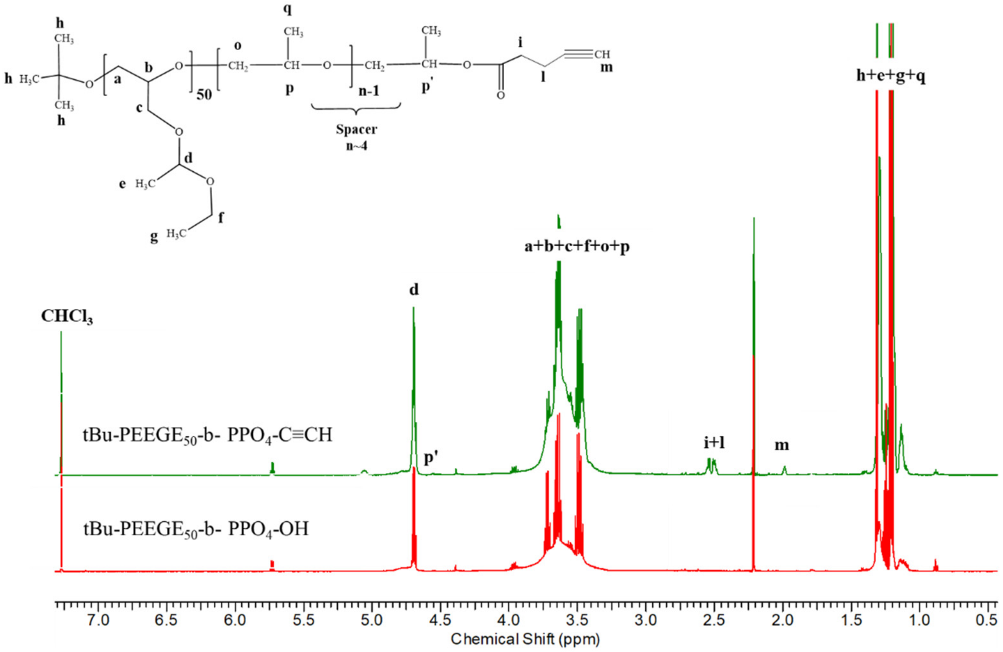

Figure S2. ^{1}H NMR spectra of tBu-PEEGE50-b-PPO4-OH and tBu-PEEGE50-b-PPO4-C≡CH in CD_3OH.

Before the modification reaction, tBu-PEEGE50-b-PPO4-OH (4.5 g, 0.5625 mmol, 1 eq) and 4-pentynoic acid (0.465 g, 4.745 mmol, 8 eq) were dried by azeotropic distillation with toluene. The dry polymer, 4-dimethyl-aminopyridine (0.1445 g, 1.184 mmol, 2 eq), and (N-(3-dimethylaminopropyl)-N'-ethylcarbodiimide hydro-chloride (0.454 g, 2.368 mmol, 4 eq) were dissolved in 20 mL of dry chloroform in a 50 mL round-bottom flask. The solution was purged with argon for 30 min, and 4-pentynoic acid dissolved in 1 mL of dry chloro-form were added dropwise. The reaction was carried out under argon at 40 °C for 120 h in the dark. The reaction mixture was filtered (0.45 μm PTFE filter) and dialyzed against a methanol/water mixture (10:1 v/v; membrane, MWCO 1000 Da) for 24 h in the dark. Then, methanol was evaporated by a rotary evaporator, and then tBu-PEEGE50-b-PPO4-C≡CH was collected by freeze-drying. The polymer was characterized by SEC (Figure S1) and ^{1}H NMR (Figure S2). Yield: 1.8 g (41%). Degree of conversion: 99 %.

S4. Synthesis and characterization data of the referent copolymer

The referent copolymer PG45-b-PCL35-b-PG45 was prepared by a click coupling reaction of α,ω-diazido func-tionalized PCL and monoalkyne functionalized PEEGE, followed by cleavage of the protective ethoxyethyl groups, as described elsewhere [48]. In brief, N_3–PCL35–N_3 (0.6031 g, 0.1507 mmol, 1 eq) and CuBr (0.4303 g, 3 mmol, 10 eq) were added to a 50 mL round-bottom flask under argon atmosphere. Dry THF (3 mL) was added via a syringe and the solution was purged with argon and stirred vigorously for 20 min. Monoalkyne-terminated PEEGE45 (1.9 g, 0.3015 mmol, 3 eq) was dissolved in dry THF (2 mL) and added to the PCL

solution along with PMDETA (0.7798 g, 4.5 mmol, 15 eq). The *click* coupling reaction was carried out at 30 °C for 24 h. The reaction mixture was cooled to RT, diluted with THF (30 mL), and filtered through a column filled with neutral alumina to remove copper complexes. The excess THF was evaporated; the crude product was dissolved in methanol (10 mL) and dialyzed against a methanol/water mixture (10:1 v/v, membrane, MWCO 8 kDa) for 72 h. The methanol was removed using a rotary vacuum evaporator, and the copolymer was recovered by freeze-drying. Yield: 1.42 g (78%). $M_{n\,GPC}$ = 15,300 g mol^{-1}, M_w/M_n = 1.8.

PEEGE blocks were derivatized into PG ones by treatment with $AlCl_3 \cdot 6H_2O$ as described elsewhere [3,4].

S5. ^{1}H NMR spectra of the novel $PEEGE_{50}$-b-PPO_4-b-[$P(CyCL)_4$-co-$(CL)_{40}$]-b-PPO_4-b-$PEEGE_{50}$ and PG_{50}-b-PPO_4-b-[$P(CyCL)_4$-co-$(CL)_{40}$]-b-PPO_4-b-PG_{50} block copolymers

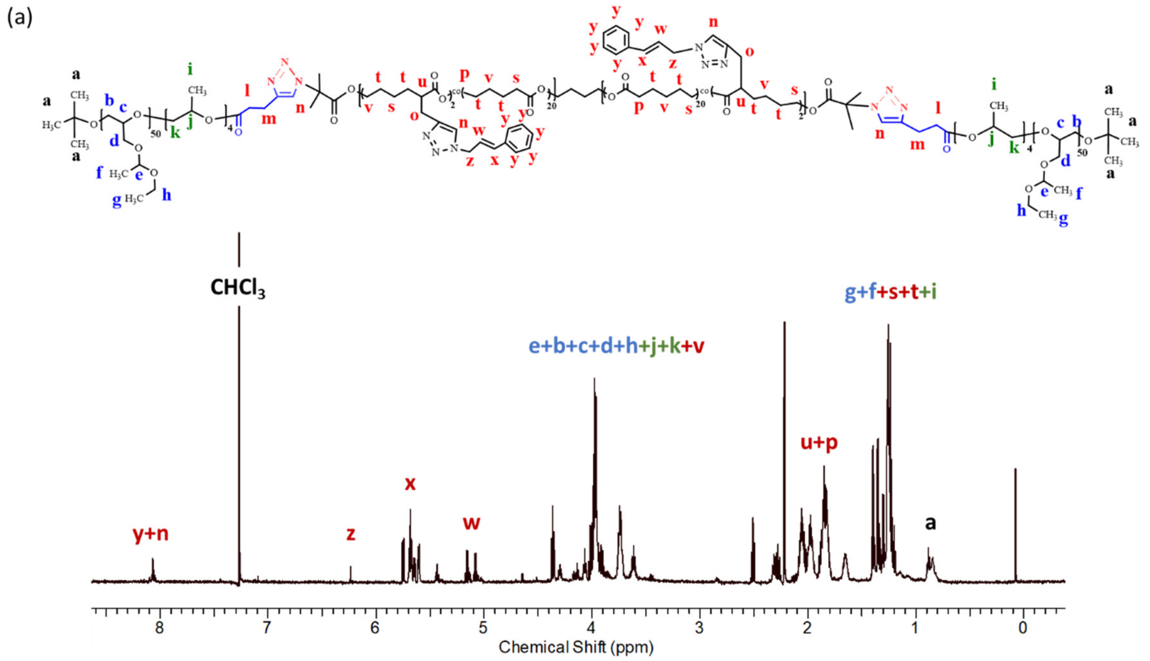

(b)

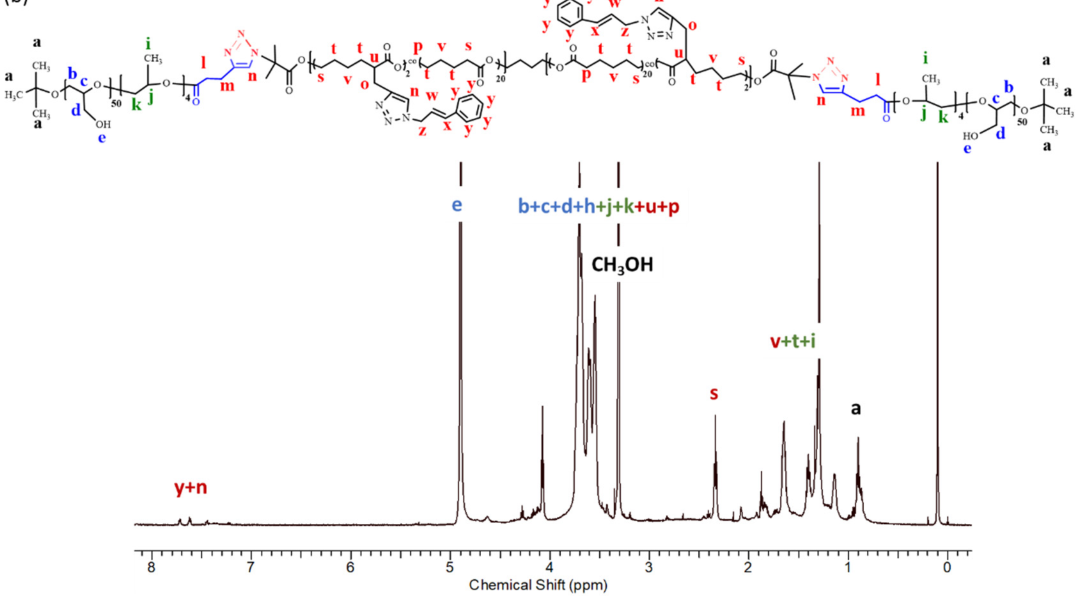

Figure S3. ^{1}H NMR spectra of (a) $PEEGE_{50}$-b-PPO_4-b-[$P(CyCL)_4$-co-$(CL)_{40}$]-b-PPO_4-b-$PEEGE_{50}$ in $CDCl_3$ and (b) PG_{50}-b-PPO_4-b-[$P(CyCL)_4$-co-$(CL)_{40}$]-b-PPO_4-b-PG_{50} in CD_3OH.

S6. Relaxation time distributions and converted therefrom particle size distributions

(a) (b)

(c) (d)

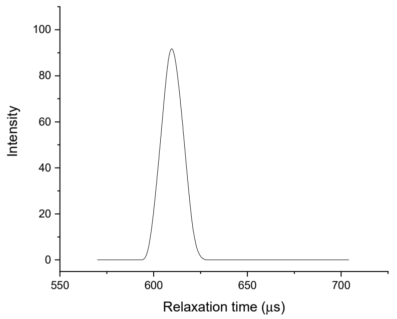

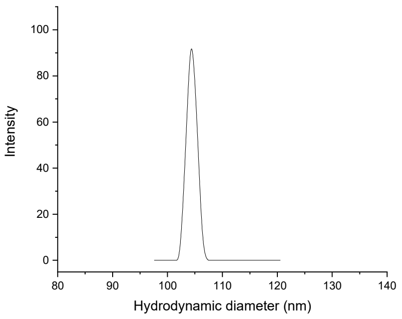

(e) (f)

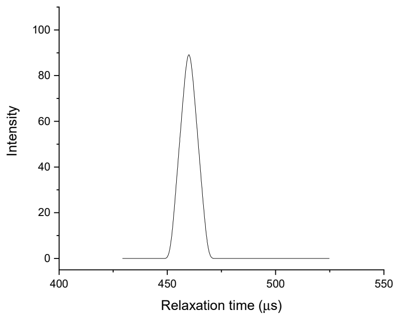

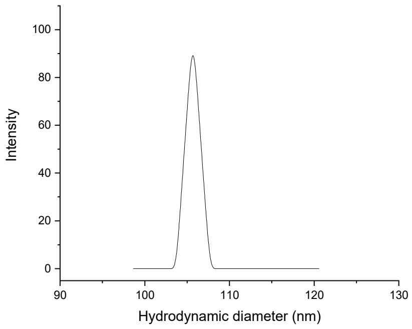

Figure S4. Relaxation time distributions (a,c,e) and the corresponding particle size distributions (b,d,f) from DLS, measured at an angle of 70° and concentration of 0.833 mg/ml (a and b), 90° and 0.714 mg/ml (c,d), and 110° and 0.417 mg/ml (e,f) for aqueous dispersions of the CBD-loaded micelles of the novel copolymer PG_{50}-b-PPO_4-b-[$P(CyCL)_4$-co-$(CL)_{40}$]-b-PPO_4-b-PG_{50}.

S7. Calculation of density of the material within the particle, ρ

ρ was calculated from the molar mass and hydrodynamic volume data assuming spherical morphology of the particles, according to equation (1).

$$\rho = \frac{3M_w}{4\pi N_A R_h^3} \quad (1)$$

Here, M_w is the weight-average molar mass of the aggregates determined by static light scattering, R_h is the hydrodynamic radius determined by dynamic light scattering, and N_A is the Avogadro's number.

S8. Light scattering characterization data of the referent copolymer micelles loaded with CBD

The CBD loaded micelles of the referent copolymer PG_{45}-b-PCL_{35}-b-PG_{45} were characterized by static light scattering. The static light scattering parameters were evaluated by the Zimm plot method. The Zimm plot is

shown in Figure S5, whereas the static parameters – weight-average molar mass (M_w), radius of gyration (R_g), and second virial coefficient (A_2) – are summarized in Table S1.

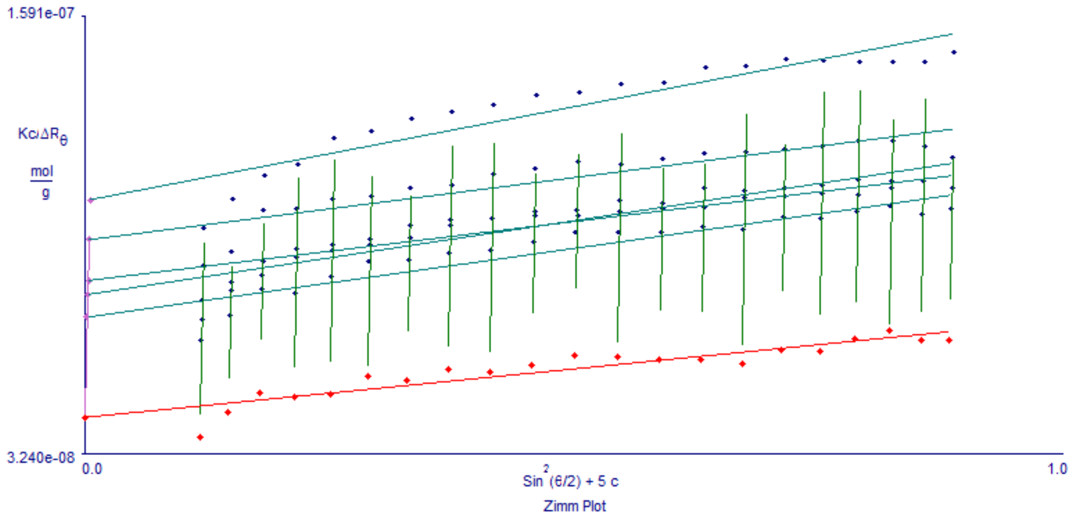

Figure S5. Zimm plot of CBD-loaded micelles of the referent copolymer in aqueous solution. Blue symbols represent experimental points. Red and purple symbols represent extrapolated points to zero concentration and zero angle, respectively. Measurements were performed at 25 °C.

Table S1. Static light scattering characterization data of the CBD-loaded micelles of the referent copolymer PG_{45}-b-PCL_{35}-b-PG_{45}. Measurements were performed at 25 °C. The standard deviations in the static light scattering parameters are up to 4%. a – expressed as number of CBD molecules loaded in one copolymer micelle.

	**10^{-6} x M_w (g/mol)**	**R_g (nm)**	**10^6 x A_2 (ml.mol/g^2)**	**Loading capacity,[a]**
**CBD-loaded referent copolymer micelles**	23.280	53.0	0.30	6170

S9. Determination of loading capacity (number of CBD molecules per micelle)

The loading capacity, expressed as number of CBD molecules per copolymer micelle of the novel copolymer, was determined as follows. From the weight-average molar mass of the CBD-loaded micelles (M_w = 26.970 x 10^6 g/mol) and total concentration (1.095 mg/ml), we determined the total number of micelles (2.23 x 10^{13}). The total number of CBD molecules (1.82x10^{17}) was determined from the molar mass and concentration of CBD, considering the encapsulation efficiency of 95 %. Assuming that all CBD molecules were loaded in the micelles, we calculated that that each micelle bore an average of 8161 CBD molecules.

Similarly, the number of CBD molecules per copolymer micelle of the referent copolymer was determined using weight-average molar mass of 23.280x10^6 g/mol, total concentration of 1.091 mg/ml, encapsulation efficiency of 91 %. From the total number of CBD molecules (1.74x10^{17}) and total number of micelles (2.82x10^{13}), assuming that all CBD molecules were loaded in the micelles, we calculated and average of 6170 CBD molecules per micelle.

S10. Determination of the Flory–Huggins parameter

The Flory–Huggins parameter χsp was calculated by the following equation:

$$\chi_{sp} = \frac{Vs\ (\ \delta s + \ \delta p)^2}{RT},$$

where Vs is the molar volume of the drug; δs and δp are the solubility parameters of the drug and the core-forming polymer, respectively; R is the gas constant; and T is the temperature in Kelvin [5]. The solubility parameters of CBD and the core-forming polymers were calculated using the contribution of the chemical groups in the molecules to their cohesive energy Table S2 [6, 7].

Table S2. Calculated values for solubility parameters (δ), drug-polymer compatibility (χsp) for PCL- and P(CyCL)-co-(CL)-containing copolymers.

**Sample**	**δ ($MPa^{1/2)}$ (Fedors method)**	χsp
**CBD**	18.65	-
**PG_{50}-b-PPO_4-b-[$P(CyCL)_4$-co-$(CL)_{40}$]-b-PPO_4-b-$PG_{50}$$PG_{45}$-b-$PCL_{35}$-b-$PG_{45}$**	18.47	0.0039
**PG_{45}-b-PCL_{35}-b-PG_{45}**	19.64	0.1195

S11. Release profiles fitting

The release profiles were fitted to different kinetic models by linear regression. Fitted release profiles of all studied compositions for the models with the best correlation are shown in Figures S6 and S7.

(a) (b)

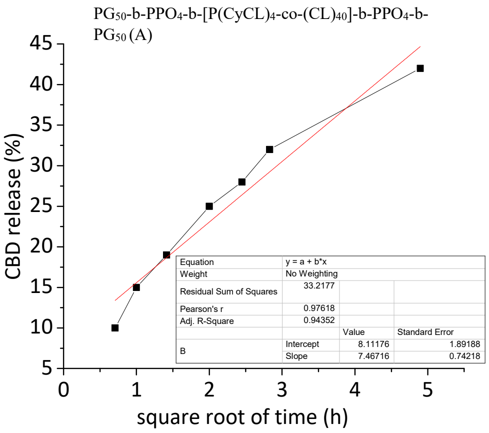

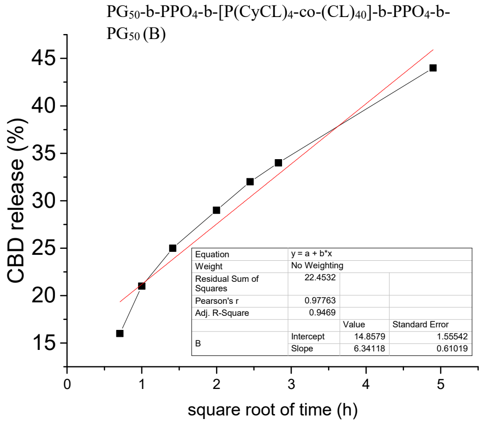

(c) (d)

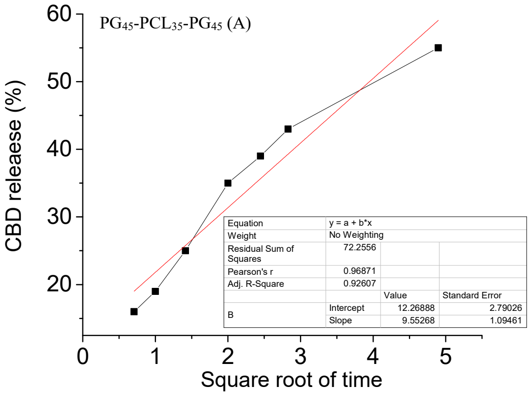

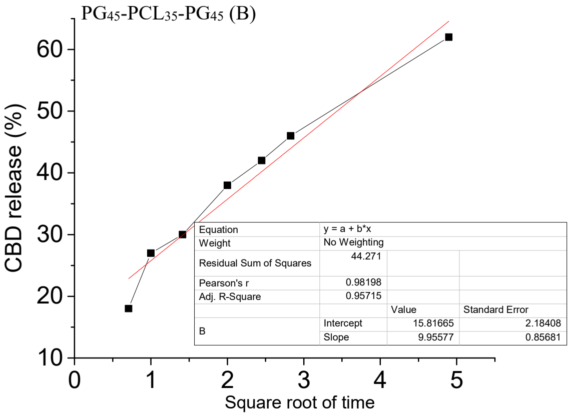

Figure S6. Release profiles of CBD from micelles of PG_{50}-b-PPO_4-b-[P(CyCL)$_4$-co-(CL)$_{40}$]-b-PPO_4-b-$PG_{50}$$PG_{45}$-b-$PCL_{35}$-b-$PG_{45}$ (a,b) and PG_{45}-b-PCL_{35}-b-PG_{45} (c,d), loaded according to Protocol A (a,c) and Protocol B (b,d) and fitting to the Higuchi kinetic model.

(a) (b)

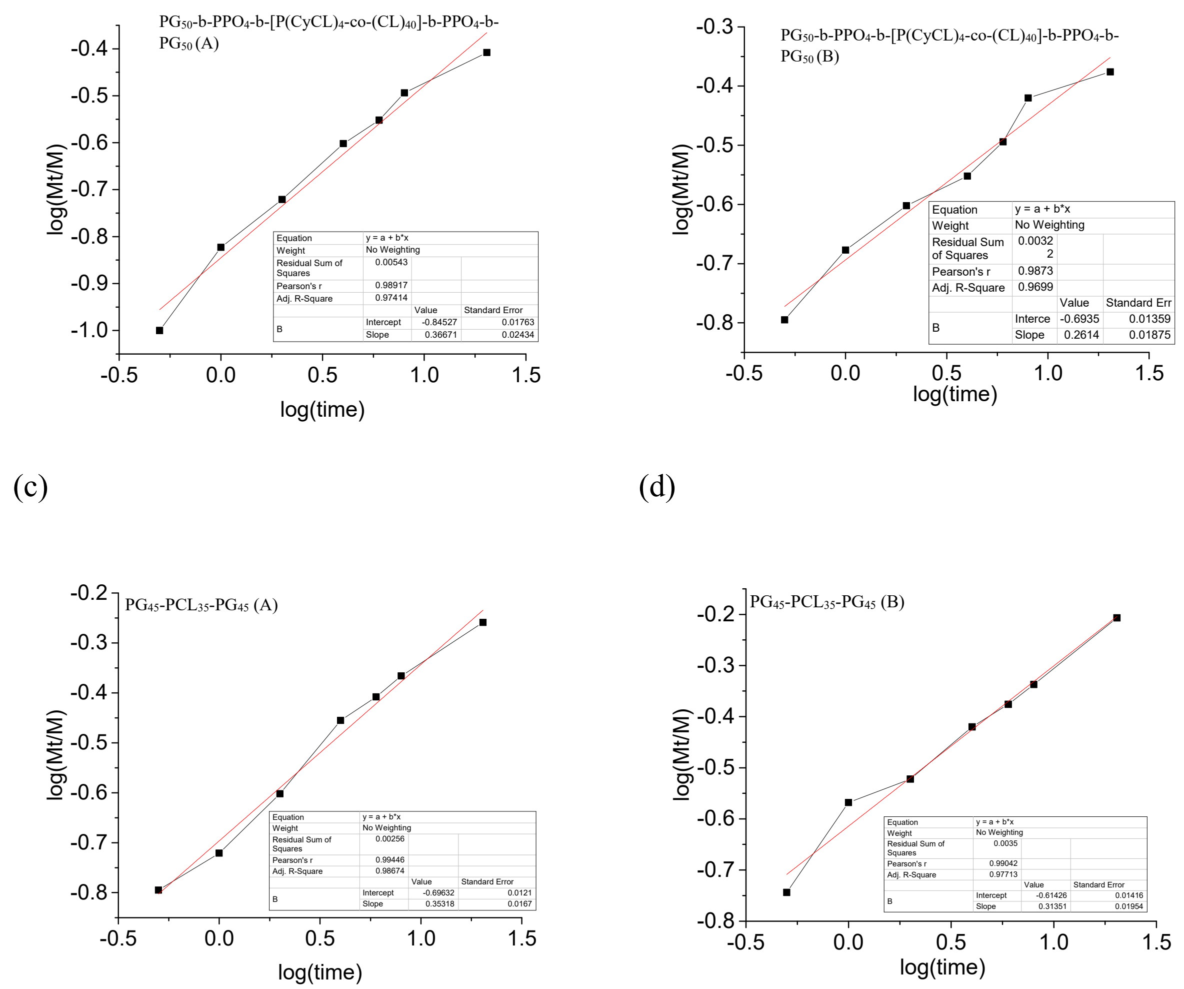

Figure S7. Release profiles of CBD from micelles of PG_{50}-b-PPO_4-b-[P(CyCL)$_4$-co-(CL)$_{40}$]-b-PPO_4-b-$PG_{50}$$PG_{45}$-b-$PCL_{35}$-b-$PG_{45}$ (a,b) and PG_{45}-b-PCL_{35}-b-PG_{45} (c,d), loaded according to Protocol A (a,c) and Protocol B (b,d) and fitting to the Korsmeyer-Peppas kinetic model.

The non-linear regression analysis whereby the drug release data were fitted to the Korsmeyer-Peppas model was carried out using DDSolver – a freely available Excel plug-in software [8]. The coefficient of determination (R^2), release rate constant (K), and diffusion exponent (n) are summarized in Table S3.

Table S3. Coefficient of determination (R^2), release rate constant (K) and diffusion exponent (n), after fitting of release profiles to Korsmeyer-Peppas kinetic model by non-linear regression analysis, using freely available Excel plug-in software DDSolver.

**Parameter**	**R^2**	**n**	**K_{KP}**

**PG_{50}-b-PPO_4-b-[P(CyCL)$_4$-co-(CL)$_{40}$]-b-PPO_4-b-PG_{50}:CBD (protocol A)**	0.971	0.304	15.622
**PG_{50}-*b*-PPO_4-*b*-[P(CyCL)$_4$-*co*-(CL)$_{40}$]-*b*-PPO_4-*b*-PG_{50} :CBD (protocol B)**	0.969	0.233	20.972
**PG_{45}-*b*-PCL_{35}-*b*-PG_{45}: CBD (protocol A)**	0.957	0.333	19.605
**PG_{45}-*b*-PCL_{35}-*b*-PG_{45}: CBD (protocol B)**	0.9908	0.298	24.990

References

[1] Vlad, R. A.; Hancu, G.; Ciurba, A.; Antonoaea. P.; Rédai, E. M.; Todoran, N.; Silasi, O.; Muntean, D. L. Cannabidiol - therapeutic and legal aspects. *Pharmazie* **2020**, *75*, 463-469. doi: 10.1691/ph.2020.0076. PMID: 33305718.

[2] Williams, C. M.; Stephens, G. J. Development of cannabidiol as a treatment for severe childhood epilepsies. *Br. J. Pharmacol.* **2020**, *177*, 5509-5517. doi: 10.1111/bph.15274.

[3] Namboodiri, V. V.; Varma, R. S. Solvent-free tetrahydropyranylation (THP) of alcohols and phenols and their regeneration by catalytic aluminum chloride hexahydrate. *Tetrahedron Lett.* **2002**, *43*, 1143−1146. https://doi.org/10.1016/S0040-4039(01)02372-3

[4] Dimitrov, P.; Rangelov, S.; Dworak, A.; Haraguchi, N.; Hirao, A.; Tsvetanov, C. B. Triblock and radial star-block copolymers comprised of poly(ethoxyethyl glycidyl ether), polyglycidol, poly(propylene oxide) and polystyrene obtained by anionic polymerization initiated by Cs initiators. *Macromol. Symp.* **2004**, *215*, 127−140. https://doi.org/10.1002/masy.200451111

[5] Flory, P. J. Principle of polymer chemistry. Ithaca, NY: Cornell University Press; 1953.

[6] Fedors, R. F. A method for estimating both the solubility parameters and molar volumes of liquids. *J. Polym. Eng. Sci.* **1974**, *14*, 147–54.

[7] Brandrup, J.; Immergut, E. H.; Grulke, E. A. Polymer Handbook, 4th Edition, Wiley, New York, 1999, pp VII-675-713.

[8] Zhang, Y.; Huo, M.; Zhou, J.; Zou, A.; Li, W.; Yao, C.; Xie, S. DDSolver: an add-in program for modeling and comparison of drug dissolution profiles. *AAPS J.* **2010**, *12 (3)*, 263-71. doi: 10.1208/s12248-010-9185-1.
